# Cost-effectiveness of one-stop-shop [^18^F]Fluorocholine PET/CT to localise parathyroid adenomas in patients suffering from primary hyperparathyroidism

**DOI:** 10.1007/s00259-024-06771-1

**Published:** 2024-06-05

**Authors:** Sietse van Mossel, Sopany Saing, Natasha Appelman-Dijkstra, Elske Quak, Abbey Schepers, Frits Smit, Lioe-Fee de Geus-Oei, Dennis Vriens

**Affiliations:** 1 Department of Radiology, Section Nuclear Medicine, Leiden University Medical Centre, Leiden, The Netherlands; 2 Biomedical Photonic Imaging, Faculty of Science and Technology, University of Twente, Enschede, The Netherlands; 3 Faculty of Behavioural Management and Social Sciences, Health Technology and Services Research, University of Twente, Enschede, The Netherlands; 4 Department of Internal Medicine, Division Endocrinology, Leiden University Medical Centre, Leiden, The Netherlands; 5 Centre for Bone Quality Leiden, Leiden University Medical Centre, Leiden, The Netherlands; 6 Department of Nuclear Medicine, Centre François Baclesse, Caen, France; 7 Department of Surgery, Leiden University Medical Centre, Leiden, The Netherlands; 8 Department of Radiology, Section Nuclear Medicine, Alrijne Medical Centre, Leiden, The Netherlands; 9 Department of Radiation Sciences and Technology, Delft University of Technology, Delft, The Netherlands; 10 Department of Medical Imaging, Radboud University Medical Centre, Nijmegen, The Netherlands

**Keywords:** Hyperparathyroidism, Parathyroid, PET/CT, Cost-effectiveness, Fluorocholine, Sestamibi

## Abstract

**Purpose:**

We conducted a cost-effectiveness analysis in which we compared a preoperative [^18^F]Fluorocholine PET/CT-based one-stop-shop imaging strategy with current best practice in which [^18^F]Fluorocholine PET/CT is only recommended after negative or inconclusive [^99m^Tc]Tc-methoxy isobutyl isonitrile SPECT/CT for patients suffering from primary hyperparathyroidism. We investigated whether the one-stop-shop strategy performs as well as current best practice but at lower costs.

**Methods:**

We developed a cohort-level state transition model to evaluate both imaging strategies respecting an intraoperative parathyroid hormone monitored treatment setting as well as a traditional treatment setting. The model reflects patients’ hospital journeys after biochemically diagnosed primary hyperparathyroidism. A cycle length of twelve months and a lifetime horizon were used. We conducted probabilistic analyses simulating 50,000 cohorts to assess joint parameter uncertainty. The incremental net monetary benefit and cost for each quality-adjusted life year were estimated. Furthermore, threshold analyses regarding the tariff of [^18^F]Fluorocholine PET/CT and the sensitivity of [^99m^Tc]Tc-methoxy isobutyl isonitrile SPECT/CT were performed.

**Results:**

The simulated long-term health effects and costs were similar for both imaging strategies. Accordingly, there was no incremental net monetary benefit and the one-stop-shop strategy did not result in lower costs. These results applied to both treatment settings. The threshold analysis indicated that a tariff of €885 for [^18^F]Fluorocholine PET/CT was required to be cost-effective compared to current best practice.

**Conclusion:**

Both preoperative imaging strategies can be used interchangeably. Daily clinical practice grounds such as available local resources and patient preferences should inform policy-making on whether a hospital should implement the one-stop-shop imaging strategy.

**Supplementary Information:**

The online version contains supplementary material available at 10.1007/s00259-024-06771-1.

## Introduction

Primary hyperparathyroidism (PHPT) is an endocrine disorder characterised by the parathyroid glands' autonomous secretion of parathyroid hormone (PTH). This is caused by a solitary parathyroid adenoma in 85% of cases, by parathyroid hyperplasia in 10% of cases, by multiple adenomas in 5% of cases and by parathyroid carcinoma in less than 1% of cases [1–4]. PHPT may result in neurological symptoms, renal events and osteoporosis with or without fractures [5, 6], causing increased morbidity and decreased quality of life (QoL) [7, 8].

The hallmark of diagnosing PHPT is biochemical testing indicating an inappropriate PTH response with respect to the serum calcium level. Imaging is used to localise the enlarged parathyroids and to determine the optimal surgical approach. In current best practice guidelines, a combination of morphological and molecular imaging is recommended consisting of cervical ultrasonography (US) and parathyroid scintigraphy with 2-phase single-photon emission computed tomography and computed tomography (SPECT/CT) using [^99m^Tc]Tc-methoxy isobutyl isonitrile (MIBI) as radiopharmaceutical [9, 10]. Currently, partial-body [^18^F]Fluorocholine ([^18^F]FCH) positron emission tomography and computed tomography (PET/CT) is only recommended after negative or inconclusive MIBI SPECT/CT [11–13]. Partial-body [^18^F]FCH PET/CT, however, might substitute conventional imaging including US and MIBI SPECT/CT [14–16], such that imaging can be developed into an [^18^F]FCH PET/CT-based one-stop-shop localisation strategy [17, 18]. In line with this, a recent network meta-analysis including a total of 8,495 patients from 119 direct competitive studies demonstrated the superior performance of [^18^F]FCH PET/CT in both patient-based and lesion-based analyses [19].

PHPT can only be cured with surgery. In case of localised single parathyroid adenoma, minimally invasive parathyroidectomy (MIP) can be performed. Otherwise, an explorative neck dissection can be performed in which all parathyroid glands are visually inspected [20–24]. To minimise the risk of persistent or recurrent hyperparathyroidism, rapid intraoperative PTH (ioPTH) monitoring can prevent a secondary neck exploration at the cost of longer durations of surgery and challenging logistics [25, 26]. MIP is associated with decreased complication rates while maintaining high cure and low recurrence rates compared to a neck exploration [27–32]. MIP also decreases the risk of hypoparathyroidism and postinterventional hospital visits [33, 34], both associated with decreased QoL [35, 36]. However, the move towards MIP highly depends on the preoperative imaging strategy used. Therefore, it is essential to evaluate the impact of imaging on long-term health effects and costs as [^18^F]FCH PET/CT is currently more expensive than MIBI SPECT/CT.

We conducted a model-based cost-effectiveness analysis in which we compared the [^18^F]FCH PET/CT one-stop-shop strategy to current best practice in which [^18^F]FCH PET/CT is only recommended after negative or inconclusive MIBI SPECT/CT. We studied whether the one-stop-shop strategy performs as well as current best practice but at a lower cost. This article aims to provide recommendations for the optimal imaging strategy to localise parathyroid adenomas when costs are taken into consideration.

## Material and methods

This study was exempt from approval by the local ethics committee as it did not include individual patient data. Instead, all model parameters were obtained from literature evidence or were elicited from an expert panel. Clinical evidence was mainly based on publications from the European Endocrine Surgical Quality Registry (EUROCRINE) including parathyroid diseases [37]. A hospital perspective was adopted in which health-related utilities and direct healthcare costs were covered. For all simulation analyses, R Statistical Software (version 4.2.1) was used [38], with software packages ‘dampack’ and ‘darthtools’ [39, 40]. This study is reported following the Consolidated Health Economic Evaluation Reporting Standards (Online Resource 1) [41].

### Imaging strategies

**Fig. 1:**
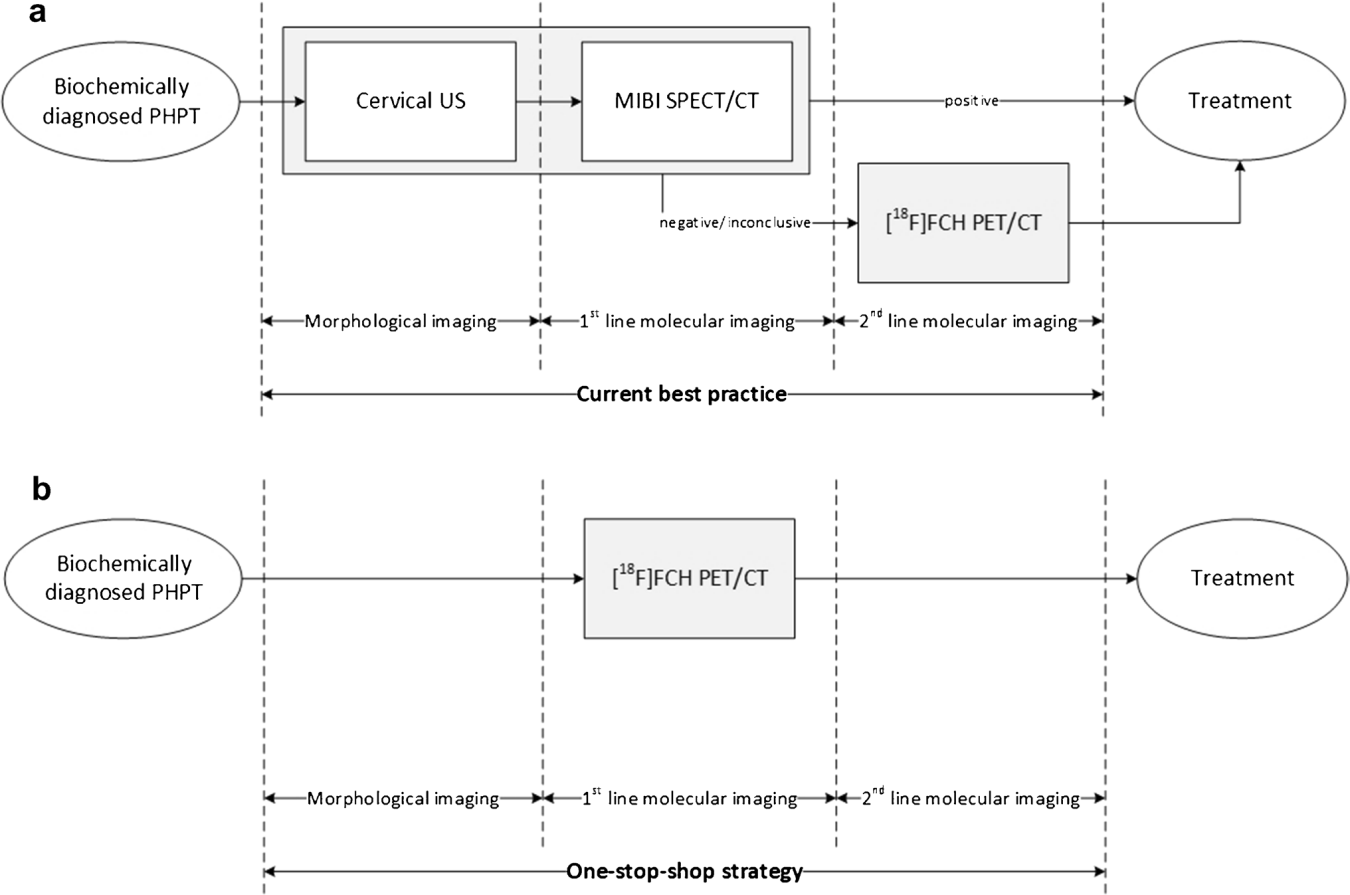
Imaging strategies are visualised. The figure shows (**a**) current best practice [9, 10] compared to (**b**) the one-stop-shop strategy [17, 18]. In current best practice, [^18^F]FCH PET/CT is only provided after negative or inconclusive MIBI SPECT/CT. In the one-stop-shop strategy, conventional imaging including US and MIBI SPECT/CT is no longer provided. Abbreviations: PHPT, primary hyperparathyroidism. US, ultrasonography. MIBI SPECT/CT, single-photon emission computed tomography and computed tomography using [^99m^Tc]Tc-methoxy isobutyl isonitrile. [^18^F]FCH PET/CT, positron emission tomography and computed tomography using [^18^F]Fluorocholine

In this study, current best practice is compared with the [^18^F]FCH PET/CT-based one-stop-shop imaging strategy. In current best practice, [^18^F]FCH PET/CT is only provided after negative or inconclusive US and MIBI SPECT/CT (Fig. 1a) [9, 10]. In the one-stop-shop strategy, conventional imaging including US and MIBI SPECT/CT will no longer be provided (Fig. 1b) [17, 18]. As described in the 2021 European Association of Nuclear Medicine practice guidelines for parathyroid imaging, preoperative US provides an additional evaluation of the thyroid that might change patient management, especially in the case of coexisting (suspected) malignant nodules [9]. Therefore, in a separate simulation, we slightly adjusted the one-stop-shop strategy by including preoperative US.

### Treatment settings

**Fig. 2:**
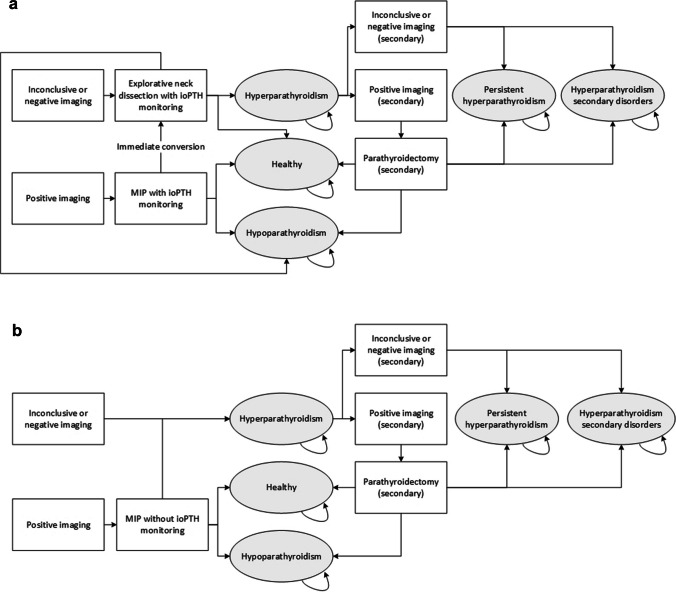
Treatment settings are visualised. The figure shows (**a**) the ioPTH-monitored treatment setting and (**b**) the traditional treatment setting. In both settings, five health states (ovals), several transition probabilities after each 12-month cycle length (arrows) and multiple lines of imaging and treatments (boxes) that patients may receive during their hospital journey are included. Patients enter the model when receiving a positive or negative/inconclusive imaging result. MIP might be directly converted to explorative neck dissection based on ioPTH monitoring. Secondary disorders include neurological, cardiovascular, bone and renal events causing increased morbidity and decreased QoL. Abbreviations: ioPTH, intraoperative parathyroid hormone serum level. MIP, minimally invasive parathyroidectomy. QoL, quality of life

We developed a cohort-level state transition model (cSTM) to evaluate the long-term health effects and costs of current best practice compared to the one-stop-shop imaging strategy. Current best practice and the one-stop-shop strategy were both covered in two different treatment settings. Both treatment settings are representative of daily clinical practice. The first treatment setting consists of MIP with ioPTH monitoring in case of positive imaging results and explorative neck dissection with ioPTH monitoring in case of negative or inconclusive imaging results, referred to as the ioPTH-monitored treatment setting (Fig. 2a). In this setting, MIP might be directly converted to explorative neck dissection based on ioPTH monitoring. The second treatment setting consists of MIP without ioPTH monitoring in case of positive imaging results and pharmacotherapy in case of negative or inconclusive imaging results, referred to as the traditional treatment setting (Fig. 2b). An expert panel consisting of two medical imaging specialists (LFG-O and DV), one endocrinologist specialised in bone and mineral diseases (NMA-D) and one surgeon specialised in endocrine neck surgery (AS), all involved in the daily care of PHPT patients, was routinely consulted to discuss the imaging strategies (Fig. 1) and corresponding treatment settings (Fig. 2).

### State-transition modelling

The cSTM reflects patients’ hospital journeys after biochemically diagnosed and localised PHPT. Separate entry points were defined for patients with positive and negative or inconclusive imaging results (Online Resource 2). Depending on the imaging result and treatment setting, patients receive MIP, explorative neck dissection, pharmacotherapy or active surveillance. MIP might be immediately converted to explorative neck dissection based on ioPTH monitoring (if accessible). Subsequently, patients are either cured, suffer from persistent hypoparathyroidism or suffer from persistent hyperparathyroidism. In case of the latter, patients have a yearly probability to receive repeat [^18^F]FCH PET/CT scans of which a subgroup will receive secondary parathyroidectomy. Otherwise, patients experience persistent hyperparathyroidism treated pharmacologically or symptomatically including active management of serum calcium concentrations and bone density with routine vitamin D supplementation and medication such as bisphosphonates, denosumab or calcimimetics. A subgroup of these patients will develop secondary disorders including neurological, cardiovascular, bone and renal events causing increased morbidity and decreased QoL.

### Model parameters

The simulated cohorts had a mean age of 62 years at baseline reflecting the mean age of patients diagnosed with PHPT [25]. A fixed cycle length of twelve months and a lifetime simulation horizon were used. Iteratively, we simulated 50,000 cohorts. The cSTM covered a large set of input parameters such as imaging characteristics, transition probabilities, relative risks, cure and mortality rates (Online Resource 3). An important parameter was the utilisation of the [^18^F]FCH PET/CT scanner in current best practice. The utilisation level directly depended on the estimated probability of negative or inconclusive conventional imaging (base case value of 20.4% of all patients [25]). Other important parameters covered the conditional probabilities of full curation after targeted and explorative parathyroidectomy (pooled base case value of 95.7% of all patients [25]) and the conditional probabilities of experiencing neurological, cardiovascular, bone or renal events in case of no curation (pooled base case value of 24.6% of all non-curative patients [5, 6]). It is also worth noting that the base case probability of immediate conversion of MIP to explorative neck dissection was 8.5% in the ioPTH-monitored treatment setting [25]. The simulated transition probabilities and relative risks were dependent on the time since the start of the simulation and the time spent in a health state. The simulation time dependency captured the increasing age-dependent background mortality and the state-residence time dependency captured the time spent in a given health state. The age-dependent background mortality rates were retrieved from Dutch demographic data for the period 2018–2022 from Statistics Netherlands [42].

### Cost information

Cost data were gathered from a healthcare perspective covering the costs related to diagnostics, medication and treatments (Online Resource 3). These costs were approximated by utilising the Dutch healthcare authority tariffs 2023 covered in the Dutch system of diagnosis treatment combinations [43] and drug database [44], accompanied by the Leiden University Medical Center specific reference tariffs. The model used a base case tariff of €965 for partial-body [^18^F]FCH PET/CT, a base case tariff of €237 for planar parathyroid scintigraphy, a base case tariff of €350 for thorax-neck MIBI SPECT/CT and a base case tariff of €84 for cervical US. The costs of treatment-related complications were reflected by covering the costs of prolonged hospitalisation and additional interventions required. According to the Dutch guidelines, an annual discount rate of 3% was applied to all future costs [45].

### Health effects

Utilities and disutilities based on EQ-5D literature evidence were gathered (Online Resource 3). The retrieved utilities reflect the valuation of health-related QoL on a scale from zero to one. The retrieved disutilities reflect the valuation of treatment-related complications [46]. Typically, cured PHPT patients will experience a better QoL (mean value of 0.84) [6] than the patients suffering from neurological, cardiovascular, bone or renal events (mean values of 0.59–0.78) [7, 8, 33]. Using these utility values, we calculated the quality-adjusted life years (QALYs) by the discounted sum of utilities over the lifetime evaluation period. According to the Dutch guidelines, an annual discount rate of 1.5% was applied to all future health outcomes [45].

### Base case analysis

Using the cSTM, we calculated the expected health effects and costs of each imaging strategy captured for each treatment setting. We expressed cost-effectiveness in terms of the net monetary benefit (NMB). The NMB was calculated by multiplying the QALYs by the willingness-to-pay (WTP) per QALY and subtracting the costs. The one-stop-shop strategy was considered cost-effective compared to current best practice when the incremental NMB was greater than zero. A WTP value of €50,000 per QALY is recommended by the Dutch healthcare authority for the expected disease burden [45].

### Probabilistic analysis

We conducted a probabilistic analysis applying Monte Carlo experiments to assess joint parameter uncertainty. We randomly sampled 50,000 parameter sets by assigning parametric distributions to all model parameters. Subsequently, both the one-stop-shop strategy and current best practice were evaluated for each parameter set. The point estimates of the cost-effectiveness ratios were plotted in cost-effectiveness planes. The incremental cost-effectiveness ratio (ICER) was the difference in costs divided by the difference in QALYs. As a function of the WTP per QALY, cost-effectiveness acceptability curves (CEACs) were used to visualise the probability that the one-stop-shop strategy was cost-effective compared to current best practice.

### Threshold analysis

We performed two threshold analyses and the results were plotted in decision curves [47]. In the first threshold analysis, we systematically decreased the base case tariff of partial-body [^18^F]FCH PET/CT until the one-stop-shop strategy led to lower costs than current best practice. Tariff estimates ranged from €600 up to €1,445 thereby opting to cover different economic European situations. Tariffs are hospital-specific as tariffs vary between Dutch hospitals ranging from estimates of €754 to €1,307 [48, 49], respectively, country-specific. For example, France handles a degressive pricing system in which tariffs vary between €550-€1,000 per scan depending on the age of the scanner and the annual number of exams performed on that scanner [50]. In the second threshold analysis, we treated the sensitivity of MIBI SPECT/CT as a continuous variable. The expected sensitivity of MIBI SPECT/CT ranged from 60%-90% since the performance of MIBI SPECT/CT is quite uncertain [14–16].

### Sensitivity analysis

To assess the robustness of the model outcomes, we performed a one-way sensitivity analysis with 95% confidence interval ranges for all model parameters compared to the base case. Furthermore, we performed sensitivity analyses in which we changed utility parameter values compared to the base case. First, we assigned a fixed disutility of 0.005 to patients receiving both MIBI SPECT/CT and [^18^F]FCH PET/CT to account for patient radiation burden [51]. Second, we assigned a fixed disutility of 0.01 to patients receiving three different preoperative scans to account for challenging logistics [11–13], which in turn might result in waiting lists, delayed localisation of the parathyroid adenomas and reduced patient satisfaction.

## Results

### Base case analysis for the ioPTH-monitored treatment setting

The expected total costs per simulated patient in the one-stop-shop strategy were similar to current best practice. The one-stop-shop strategy had an estimated mean total cost of €3,841 per patient and current best practice had an estimated mean total cost of €3,822 per patient. Also, there was no expected clinically relevant difference in the QALYs obtained as the estimated mean total QALY was 12.65 per patient in both strategies. Consequently, the incremental NMB was approximately zero. The results of the Monte Carlo experiments were plotted in cost-effectiveness planes (Online Resource 4, Fig. 1). The results of the adjusted one-stop-shop strategy including preoperative US were reported in Online Resource 5.

### Base case analysis for the traditional treatment setting

The simulation outcomes of the traditional treatment setting were in line with the ioPTH-monitored treatment setting. The expected total costs per simulated patient in the one-stop-shop strategy were similar to current best practice. The one-stop-shop strategy had an estimated mean total cost of €4,535 per patient and current best practice had an estimated mean total cost of €4,514 per patient. There was no expected clinically relevant difference in the QALYs obtained as the estimated mean total QALY was 12.64 per patient in both strategies. Consequently, the incremental NMB was again approximately zero. The results of the Monte Carlo experiments were plotted in cost-effectiveness planes (Online Resource 4, Fig. 1). The results of the adjusted one-stop-shop strategy including preoperative US were reported in Online Resource 5.

### Threshold analysis

**Fig. 3:**
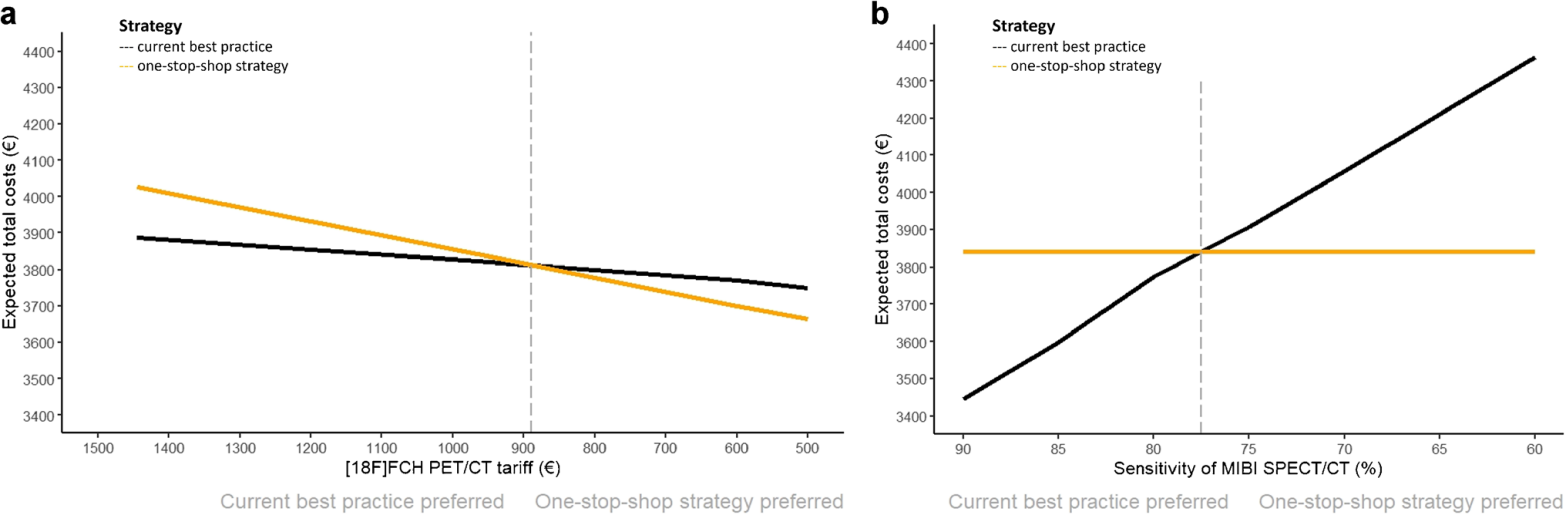
Decision curves depicting **(a)** the tariff of [^18^F]FCH PET/CT on the x-axis and **(b)** the sensitivity of MIBI SPECT/CT on the x-axis. The total costs including imaging, surgery and pharmacotherapy of the expected PHPT care pathway are depicted on the y-axis and given an ioPTH-monitored treatment setting. Abbreviations: ioPTH, intraoperative parathyroid hormone serum level. [^18^F]FCH PET/CT, positron emission tomography and computed tomography using [^18^F]Fluorocholine. MIBI SPECT/CT, single-photon emission computed tomography and computed tomography using [^99m^Tc]Tc-methoxy isobutyl isonitrile. PHPT, primary hyperparathyroidism

Decision curves show the results of the threshold analyses for the ioPTH-monitored treatment setting. Figure 3a shows that the one-stop-shop strategy was cost-effective when the base case tariff of partial-body [^18^F]FCH PET/CT (€965) decreased by at least €80 (8.3%) resulting in a suggested tariff of €885. Figure 3b shows that the one-stop-shop strategy was cost-effective when the base case sensitivity of MIBI SPECT/CT (79.6%) decreased to sensitivity values lower than 78%. The results of the threshold analyses for the traditional treatment setting were similar (Online Resource 4, Fig. 2).

### Sensitivity analysis

**Fig. 4:**
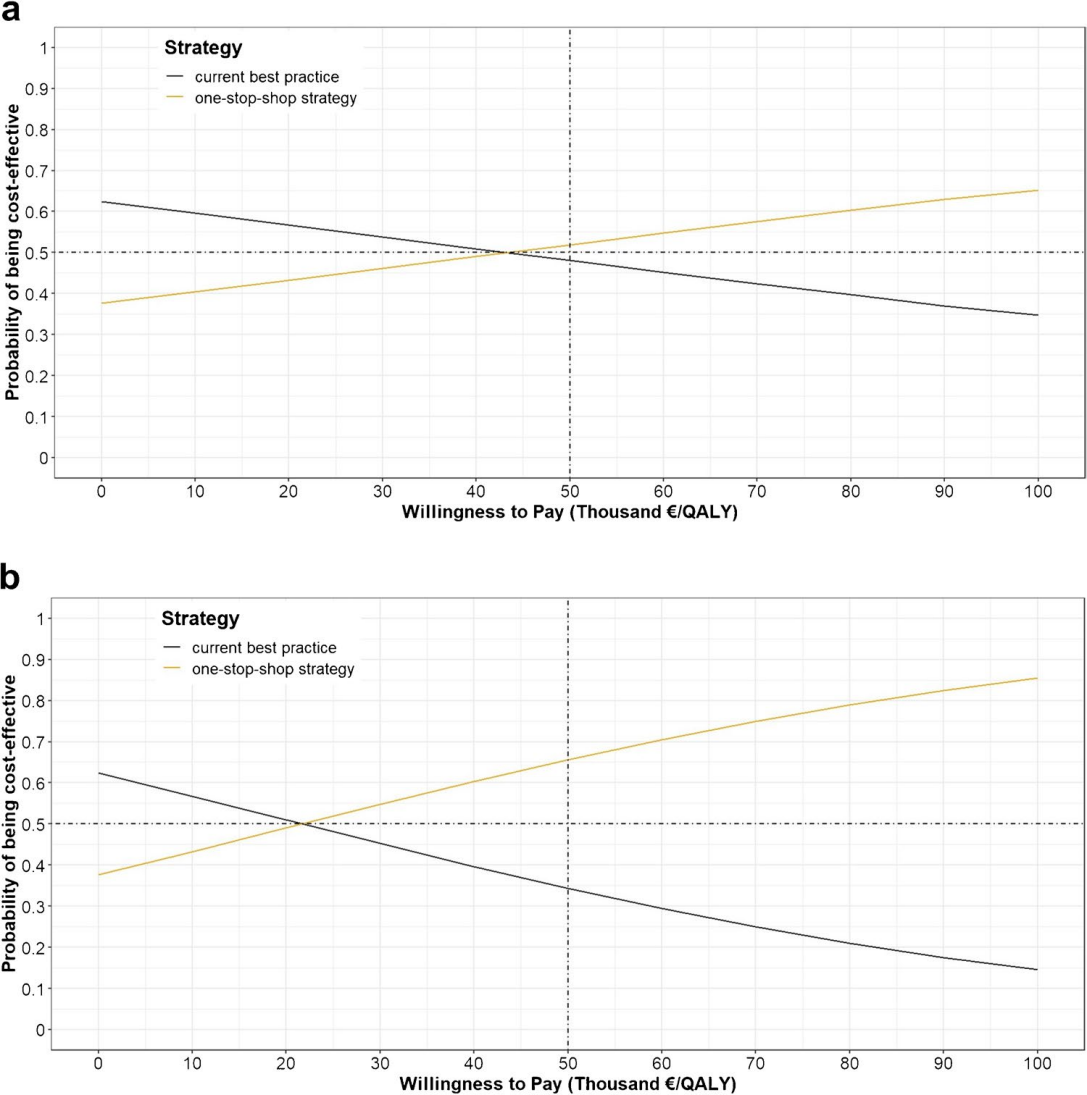
The cost-effectiveness acceptability curves show the probability that the one-stop-shop strategy is cost-effective compared to current best practice at different WTP thresholds per QALY ranging from €0 to €100,000. The first curve (**a**) shows that incorporating a fixed disutility of 0.005, as a consequence of radiation burden for patients receiving both MIBI SPECT/CT and [^18^F]FCH PET/CT, results in an ICER of €48,092 and a 52% probability that the one-stop-shop strategy is cost-effective at the most accepted WTP threshold of €50,000 per QALY. The second curve (**b**) shows that incorporating a fixed disutility of 0.01, as a consequence of challenging logistics for patients receiving three different preoperative scans, results in an ICER of €24,046 and a 66% probability that the one-stop-shop strategy is cost-effective at the most accepted WTP threshold of €50,000 per QALY. Abbreviations: WTP, willingness-to-pay. QALY, quality-adjusted life year. ICER, incremental cost-effectiveness ratio. [^18^F]FCH PET/CT, positron emission tomography and computed tomography using [^18^F]Fluorocholine. MIBI SPECT/CT, single-photon emission computed tomography and computed tomography using [^99m^Tc]Tc-methoxy isobutyl isonitrile

A tornado diagram of the one-way sensitivity analysis visualises the input parameters that led to a relative change in the incremental NMB of at least 10% compared to the base case (Online Resource 4, Fig. 3). We observed that the tariff of partial-body [^18^F]FCH PET/CT had the most impact on the incremental NMB. Furthermore, we applied disutilities to model the health consequences of patient radiation burden and challenging logistics. The corresponding CEACs were plotted for the ioPTH-monitored treatment setting. If incorporating a disutility for radiation burden, the expected ICER was €48,092 and the probability of the one-stop-shop strategy being cost-effective was 52% at the most accepted WTP threshold of €50,000 per QALY (Fig. 4a). If incorporating a disutility for challenging logistics, the expected ICER was €24,046 and the probability of the one-stop-shop strategy being cost-effective was 66% at the most accepted WTP threshold of €50,000 per QALY (Fig. 4b). The results of sensitivity analyses for the traditional treatment setting were similar (Online Resource 4, Fig. 4).

## Discussion

This study explored the cost-effectiveness of two imaging strategies to localise parathyroid adenomas and to guide succeeding treatment for patients suffering from PHPT: the [^18^F]FCH PET/CT-based one-stop-shop strategy and current best practice in which [^18^F]FCH PET/CT is only recommended after negative or inconclusive MIBI SPECT/CT. The simulated health effects and costs were similar for both imaging strategies. Accordingly, the one-stop-shop strategy is not associated with lower costs and both imaging strategies can be used interchangeably. This applies to both the ioPTH-monitored treatment setting in which MIP might immediately be converted to explorative neck dissection as well as the traditional treatment setting in which ioPTH is not monitored. The total PHPT care pathway costs, however, varied between the ioPTH-monitored and traditional treatment setting (estimated values of €3,822-€3,841 compared to €4,514-€4,535). A recent Cochrane review discussed that there exists considerable uncertainty surrounding such estimates which might be explained by differences in hospital charges for surgical procedures as well as differences in the surgical protocols [26].

In essence, the decision to implement either imaging strategy depends on available local resources as well as patient preferences. We want to emphasise that daily clinical practice grounds – such as easy resource capacity allocation, reduced waiting times, reduced travel times, reduced hospital waste, reduced patient radiation burden and meeting patient preferences – should inform the policy-making on whether a hospital should implement the one-stop-shop imaging strategy. The one-stop-shop strategy decreases the number of hospital visits which is fundamentally preferable in terms of logistics, environmental impact and interference in patients’ lives. Also, the one-stop-shop strategy is preferred when healthcare resources are limited as it has a lower impact on scarce resources. Moreover, the one-stop-shop strategy might save approximately half the radiation burden of current best practice. Of course, the availability of [^18^F]FCH PET/CT scanners, radiopharmaceuticals and personnel should be taken into consideration when opting for the one-stop-shop strategy.

In current practice, preoperative cervical US is used to rule out thyroid disorders [9]. Nevertheless, a strategy including US and [^18^F]FCH PET/CT but without MIBI SPECT/CT might not be considered a relevant strategy for cost-effectiveness analysis. Most importantly, because [^18^F]FCH PET/CT allows for MIP, therefore, trauma after surgery is generally minimal and does not lead to additional risks if re-intervention in the neck region (for thyroid disease) would be necessary. Second, because [^18^F]FCH PET/CT has a very high negative predictive value to rule out thyroid cancer [52], and the low-dose CT performed during [^18^F]FCH PET/CT may not be suitable to exclude coincidental thyroid carcinoma but provides the surgeon with anatomical context in case of thyroid nodules or goitre. If the local situation does not allow omitting US, costs will increase approximately at the rate of preoperative US but a clinically relevant increase in QALYs may not be expected (Online Resource 5).

To our knowledge, only one study assessed the cost-effectiveness of [^18^F]FCH PET/CT regarding the localisation of enlarged parathyroids [53]. The authors argued that [^18^F]FCH PET/CT is potentially a cost-effective imaging technique in the United States. They, however, performed a head-to-head comparison of [^18^F]FCH PET/CT with stand-alone conventional imaging including 4-dimensional CT, US, and MIBI SPECT, while we compared an [^18^F]FCH PET/CT-based one-stop-shop strategy with the use of [^18^F]FCH PET/CT after negative or inconclusive conventional imaging including sequential US and MIBI SPECT/CT. Where models focus on stand-alone imaging without consideration of sequential imaging, there is a profound source of structural uncertainty implying that the cost-effectiveness of imaging across the PHPT care pathway remains uncertain [54–56].

The American study reported substantial incremental QALY benefits (values of 0.02–0.05) [53], whereas we could not find such QALY benefits. The American study also reported relatively high cost outcomes (values approximating €10,000) [53], whereas we found at most half these costs. These differences might be explained by the modelling methods used in combination with the different tariffs of imaging, surgery and pharmacotherapy within the American and European healthcare systems. The choice of modelling method depends on the complexity of the clinical process to be modelled, the available evidence and the modellers’ experience. We obtained all model parameter values from aggregated literature evidence complemented with expert elicitation, justifying that we developed a cSTM.

This study has some limitations. We simulated imaging test results using conditional probabilities reflecting the diagnostic performance of MIBI SPECT/CT and [^18^F]FCH PET/CT. The performance of [^18^F]FCH PET/CT, however, varies between current best practice and the one-stop-shop strategy because [^18^F]FCH PET/CT can be used as first-line or second-line imaging. Where there was abundant evidence available to properly estimate the performance of [^18^F]FCH PET/CT as second-line imaging (base case accuracy of 76% used for simulations [11–13]), limited evidence was available of [^18^F]FCH PET/CT as first-line imaging (base case accuracy of 96% used for simulations [14–16]). Nonetheless, [^18^F]FCH PET/CT would be the most favourable imaging technique for the localisation of parathyroid adenomas and its superior performance has been demonstrated in several reviews and meta-analyses [19, 57–61]. Sequentially, patient outcomes can be improved with effective patient management after localisation with imaging. Further patient management depends on contextual factors within the treatment pathway including the availability of surgical and pharmacological procedures and their effectiveness. Therefore, assessing the clinical utility of imaging requires an integrated approach that considers all contextual factors along the treatment pathway.

Also, the time to repeat imaging may impact clinical utility. The simulated time to repeat imaging was based on expert judgements assuming a constant exponential hazard rate as we tend to decrease imaging frequency once the previous imaging results were negative. We validated whether this assumption was accurate with extreme parameter value testing, testing of traces in which patients were tracked through the model, and unit testing in which sub-modules of the model were tested. The time to repeat imaging was found to be comparable with published EUROCRINE evidence [37].

It is noteworthy that the clinical utility of imaging might be estimated in randomised controlled trials. However, trials are not always recommended since they are costly and time-consuming. They are also considered less feasible given all combinations of imaging and therapeutics that can be compared in this setting. Moreover, trials do often not allow assessment of lifetime outcomes or risks related to imaging (e.g., patient radiation burden due to imaging). Model-based cost-effectiveness analysis should be seen as a valid alternative [62]. However, cost-effectiveness models evaluating imaging will be more complex and require more evidence than models evaluating therapeutics. Given the complexity and dependencies related to the use of imaging, researchers may not always be fully aware of all the different aspects potentially influencing the results of a model-based cost-effectiveness study, and the results of model-based cost-effectiveness studies are only relevant insofar as they represent current clinical practice in the specific decision context [63].

The results of this study can be used to revise international guidelines for parathyroid imaging [9, 10]. The need for revision is underpinned by previous studies showing that there exists a substantial variation in the imaging tests used [17, 18]. Additional studies, preferably based on data from randomised clinical trials and analysing clinical patterns across multiple lines of sequential imaging rather than stand-alone imaging, are required to reach a comprehensive evidence base for guideline improvement. The inclusion of cost-effectiveness studies in the guideline revision process will lead to a more sensible use of scarce healthcare resources.

This study represents the first cost-effectiveness analysis encompassing sequential imaging, surgery and pharmacotherapy. We demonstrated that the one-stop-shop imaging strategy can be seamlessly integrated into routine clinical practice with negligible additional expenses for hospitals. Therefore, the adoption of the one-stop-shop strategy hinges primarily on the availability of local resources.

## Supplementary Information

Below is the link to the electronic supplementary material.

Supplementary file1 (PDF 209 KB)

Supplementary file2 (PDF 156 KB)

Supplementary file3 (PDF 295 KB)

Supplementary file4 (PDF 712 KB)

Supplementary file5 (PDF 786 KB)

## Data Availability

The datasets generated and/or analysed during the current study are available in the Supplementary Information. Software codes on which the conclusions of our paper rely are available upon reasonable substantiated request.
