# Supplementary material for: Cost-effectiveness of one-stop-shop [18F]Fluorocholine PET/CT to localise parathyroid adenomas in patients suffering from primary hyperparathyroidism
Source: Eur J Nucl Med Mol Imaging. 2024 Jun 5;51(12):3585–95. doi: 10.1007/s00259-024-06771-1 (PMC11457719; doi:10.1007/s00259-024-06771-1)
Supplement: Supplementary file 1 — Supplementary file1 (PDF 209 KB) [file 259_2024_6771_MOESM1_ESM.pdf]

# Cost-Effectiveness of One-Stop-Shop [$^{18}$F]Fluorocholine PET/CT to Localise Parathyroid Adenomas in Patients Suffering from Primary Hyperparathyroidism

*European Journal of Nuclear Medicine and Molecular Imaging (EJNMMI)*

Sietse van Mossel [1,2,*], Sopany Saing [3], Natasha Appelman-Dijkstra [4,5], Elske Quak [6], Abbey Schepers [7], Frits Smit[1,8], Lioe-Fee de Geus-Oei [1,2,9], Dennis Vriens [1,5,10]

[1] Department of Radiology, section Nuclear Medicine, Leiden University Medical Centre, Leiden, The Netherlands

[2] Biomedical Photonic Imaging, Faculty of Science and Technology, University of Twente, Enschede, The Netherlands

[3] Health Technology and Services Research, Faculty of Behavioural Management and Social Sciences, University of Twente, Enschede, The Netherlands

[4] Department of Internal Medicine, division Endocrinology, Leiden University Medical Centre, Leiden, The Netherlands

[5] Centre for Bone Quality Leiden, Leiden University Medical Centre, Leiden, The Netherlands

[6] Department of Nuclear Medicine, Centre François Baclesse, Caen, France

[7] Department of Surgery, Leiden University Medical Centre, Leiden, The Netherlands

[8] Department of Radiology, section Nuclear Medicine, Alrijne Medical Centre, Leiden, The Netherlands

[9] Department of Radiation Sciences and Technology, Delft University of Technology, Delft, The Netherlands

[10] Department of Medical Imaging, Radboud University Medical Centre, Nijmegen, The Netherlands

* Corresponding author: Ir. Sietse van Mossel (s.van_mossel@lumc.nl; 2333 ZA Leiden, The Netherlands)

Consolidated Health Economic Evaluation Reporting Standards 2022 checklist [1]

| Topic | No. | Item | Location where item is reported |
|---|---|---|---|
| **Title** | | | |
| | 1 | Identify the study as an economic evaluation and specify the interventions being compared. | Title |
| **Abstract** | | | |
| | 2 | Provide a structured summary that highlights context, key methods, results, and alternative analyses. | Abstract |
| **Introduction** | | | |
| **Background and objectives** | 3 | Give the context for the study, the study question, and its practical relevance for decision making in policy or practice. | Introduction |
| **Methods** | | | |
| **Health economic analysis plan** | 4 | Indicate whether a health economic analysis plan was developed and where available. | Methods, Paragraph "State-transition modelling" |
| **Study population** | 5 | Describe characteristics of the study population (such as age range, demographics, socioeconomic, or clinical characteristics). | Methods, Paragraph "Treatment settings" |
| **Setting and location** | 6 | Provide relevant contextual information that may influence findings. | Methods, Paragraph "Treatment settings" |
| **Comparators** | 7 | Describe the interventions or strategies being compared and why chosen. | Methods, Paragraph "Imaging strategies" |
| **Perspective** | 8 | State the perspective(s) adopted by the study and why chosen. | Methods, First Paragraph |

| Topic | No. | Item | Location where item is reported |
|---|---|---|---|
| **Time horizon** | 9 | State the time horizon for the study and why appropriate. | Methods, Paragraph "Model parameters" |
| **Discount rate** | 10 | Report the discount rate(s) and reason chosen. | Methods, Paragraphs "Cost information" and "Health effects" |
| **Selection of outcomes** | 11 | Describe what outcomes were used as the measure(s) of benefit(s) and harm(s). | Methods, Paragraph "Base case analysis" |
| **Measurement of outcomes** | 12 | Describe how outcomes used to capture benefit(s) and harm(s) were measured. | Methods, Paragraph "Base case analysis" |
| **Valuation of outcomes** | 13 | Describe the population and methods used to measure and value outcomes. | Methods, Paragraph "Base case analysis" |
| **Measurement and valuation of resources and costs** | 14 | Describe how costs were valued. | Methods, Paragraphs "Cost information" and "Health effects" |
| **Currency, price date, and conversion** | 15 | Report the dates of the estimated resource quantities and unit costs, plus the currency and year of conversion. | Methods, Paragraph "Cost information" |
| **Rationale and description of model** | 16 | If modelling is used, describe in detail and why used. Report if the model is publicly available and where it can be accessed. | Methods, Paragraph "State-transition modelling" and Declarations |
| **Analytics and assumptions** | 17 | Describe any methods for analysing or statistically transforming data, any extrapolation methods, and approaches for validating any model used. | Methods , Paragraph "Model parameters" and Appendix |

| Topic | No. | Item | Location where item is reported |
|---|---|---|---|
| **Characterising heterogeneity** | 18 | Describe any methods used for estimating how the results of the study vary for subgroups. | Methods , Paragraphs "Probabilistic analysis", "Sensitivity analysis" and "Threshold analysis" |
| **Characterising distributional effects** | 19 | Describe how impacts are distributed across different individuals or adjustments made to reflect priority populations. | Methods, Paragraph "Probabilistic analysis" |
| **Characterising uncertainty** | 20 | Describe methods to characterise any sources of uncertainty in the analysis. | Methods, Paragraph "Probabilistic analysis" |
| **Approach to engagement with patients and others affected by the study** | 21 | Describe any approaches to engage patients or service recipients, the general public, communities, or stakeholders (such as clinicians or payers) in the design of the study. | Not reported |
| **Results** | | | |
| **Study parameters** | 22 | Report all analytic inputs (such as values, ranges, references) including uncertainty or distributional assumptions. | Appendix |
| **Summary of main results** | 23 | Report the mean values for the main categories of costs and outcomes of interest and summarise them in the most appropriate overall measure. | Results, first and second paragraph |
| **Effect of uncertainty** | 24 | Describe how uncertainty about analytic judgments, inputs, or projections affect findings. Report the effect of choice of discount rate and time horizon, if applicable. | Results, third and fourth paragraph |

| Topic | No. | Item | Location where item is reported |
|---|---|---|---|
| **Effect of engagement with patients and others affected by the study** | 25 | Report on any difference patient/service recipient, general public, community, or stakeholder involvement made to the approach or findings of the study | Not reported |
| **Discussion** | | | |
| **Study findings, limitations, generalisability, and current knowledge** | 26 | Report key findings, limitations, ethical or equity considerations not captured, and how these could affect patients, policy, or practice. | Discussion |
| **Other relevant information** | | | |
| **Source of funding** | 27 | Describe how the study was funded and any role of the funder in the identification, design, conduct, and reporting of the analysis | Statements and Declarations |
| **Conflicts of interest** | 28 | Report authors conflicts of interest according to journal or International Committee of Medical Journal Editors requirements. | Statements and Declarations |

## References

1. Husereau D, Drummond M, Augustovski F, De Bekker-Grob E, Briggs AH, Carswell C, et al. Consolidated Health Economic Evaluation Reporting Standards 2022 (CHEERS 2022) statement: updated reporting guidance for health economic evaluations. BMJ. 2022;376:e067975.
