# Supplementary material for: Cost-effectiveness of one-stop-shop [18F]Fluorocholine PET/CT to localise parathyroid adenomas in patients suffering from primary hyperparathyroidism
Source: Eur J Nucl Med Mol Imaging. 2024 Jun 5;51(12):3585–95. doi: 10.1007/s00259-024-06771-1 (PMC11457719; doi:10.1007/s00259-024-06771-1)
Supplement: Supplementary file 2 — Supplementary file2 (PDF 156 KB) [file 259_2024_6771_MOESM2_ESM.pdf]

Cost-Effectiveness of One-Stop-Shop [^{18}F]Fluorocholine PET/CT to Localise Parathyroid Adenomas in Patients Suffering from Primary Hyperparathyroidism

European Journal of Nuclear Medicine and Molecular Imaging (EJNMMI)

Sietse van Mossel [1,2,*], Sopany Saing [3], Natasha Appelman-Dijkstra [4,5], Elske Quak [6], Abbey Schepers [7], Frits Smit[1,8], Lioe-Fee de Geus-Oei [1,2,9], Dennis Vriens [1,5,10]

[1] Department of Radiology, section Nuclear Medicine, Leiden University Medical Centre, Leiden, The Netherlands

[2] Biomedical Photonic Imaging, Faculty of Science and Technology, University of Twente, Enschede, The Netherlands

[3] Health Technology and Services Research, Faculty of Behavioural Management and Social Sciences, University of Twente, Enschede, The Netherlands

[4] Department of Internal Medicine, division Endocrinology, Leiden University Medical Centre, Leiden, The Netherlands

[5] Centre for Bone Quality Leiden, Leiden University Medical Centre, Leiden, The Netherlands

[6] Department of Nuclear Medicine, Centre François Baclesse, Caen, France

[7] Department of Surgery, Leiden University Medical Centre, Leiden, The Netherlands

[8] Department of Radiology, section Nuclear Medicine, Alrijne Medical Centre, Leiden, The Netherlands

[9] Department of Radiation Sciences and Technology, Delft University of Technology, Delft, The Netherlands

[10] Department of Medical Imaging, Radboud University Medical Centre, Nijmegen, The Netherlands

* Corresponding author: Ir. Sietse van Mossel (s.van_mossel@lumc.nl; 2333 ZA Leiden, The Netherlands)

Tree-based structures of the preoperative imaging workups

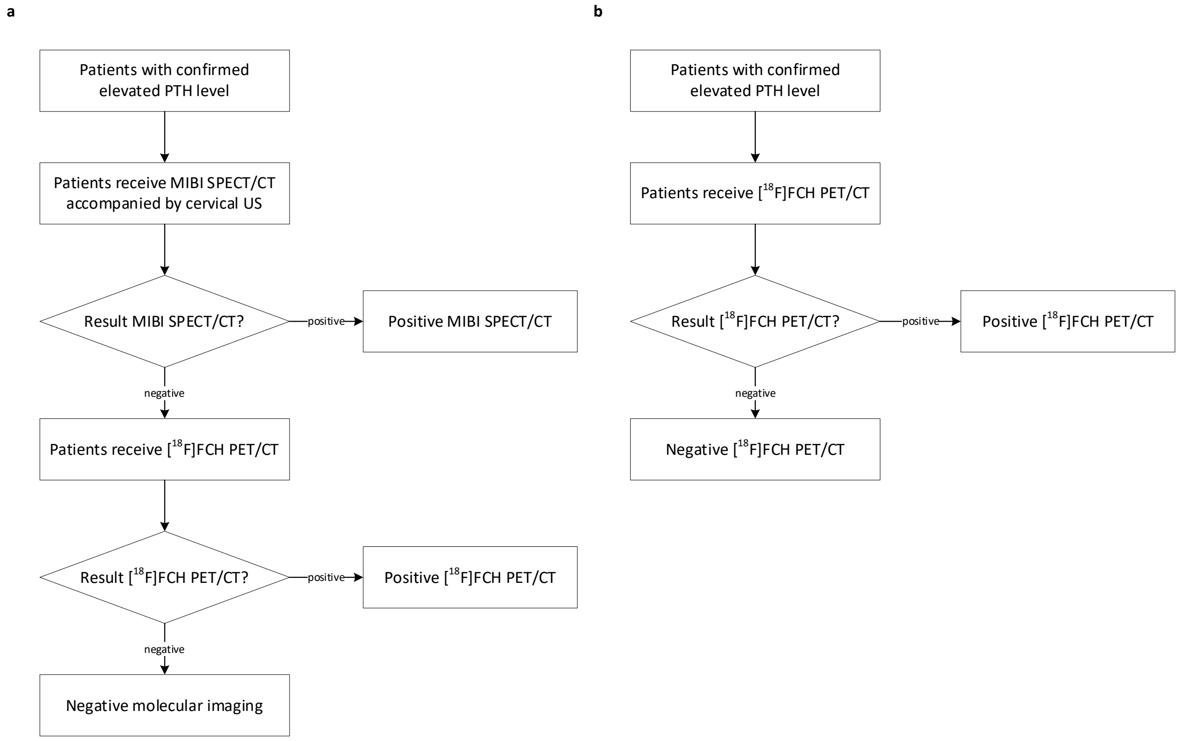

Tree-based structures reflecting the preoperative imaging workups are visualised. The visualisation shows (**a**) current best practice based on [1,2] compared to (**b**) the one-stop-shop strategy based on [3,4]. In current best practice, [^{18}F]FCH PET/CT is only provided in case of a negative or inconclusive MIBI SPECT/CT. In the one-stop-shop strategy, conventional imaging including US and MIBI SPECT/CT is no longer provided. Abbreviations: PTH, parathyroid hormone. US, ultrasonography. MIBI SPECT/CT, single-photon emission computed tomography and computed tomography using [^{99m}Tc]Tc-methoxy isobutyl isonitrile. [^{18}F]FCH PET/CT, positron emission tomography and computed tomography using [^{18}F]Fluorocholine.

References

1. Ovčariček PP, Giovanella L, Gasset IC, Hindié E, Huellner MW, Luster M, et al. The EANM practice guidelines for parathyroid imaging. Eur J Nucl Med Mol Imaging. 2021;48:2801–22.

2. Hindié E, Ugur Ö, Fuster D, O'Doherty M, Grassetto G, Ureña P, et al. 2009 EANM parathyroid guidelines. Eur J Nucl Med Mol Imaging. 2009;36:1201–16.

3. Hindi E, Schwartz P, Avram AM, Imperiale A, Sebag F, Taïeb D. Primary Hyperparathyroidism: Defining the Appropriate Preoperative Imaging Algorithm. J Nucl Med. 2021;62:3–12.

4. Giovanella L, Bacigalupo L, Treglia G, Piccardo A. Will 18 F-fluorocholine PET/CT replace other methods of preoperative parathyroid imaging? Endocrine. 2020;71:285–97.
