# Supplementary material for: Cost-effectiveness of one-stop-shop [18F]Fluorocholine PET/CT to localise parathyroid adenomas in patients suffering from primary hyperparathyroidism
Source: Eur J Nucl Med Mol Imaging. 2024 Jun 5;51(12):3585–95. doi: 10.1007/s00259-024-06771-1 (PMC11457719; doi:10.1007/s00259-024-06771-1)
Supplement: Supplementary file 3 — Supplementary file3 (PDF 295 KB) [file 259_2024_6771_MOESM3_ESM.pdf]

Cost-Effectiveness of One-Stop-Shop [^{18}F]Fluorocholine PET/CT to Localise Parathyroid Adenomas in Patients Suffering from Primary Hyperparathyroidism

European Journal of Nuclear Medicine and Molecular Imaging (EJNMMI)

Sietse van Mossel [1,2,*], Sopany Saing [3], Natasha Appelman-Dijkstra [4,5], Elske Quak [6], Abbey Schepers [7], Frits Smit[1,8], Lioe-Fee de Geus-Oei [1,2,9], Dennis Vriens [1,5,10]

[1] Department of Radiology, section Nuclear Medicine, Leiden University Medical Centre, Leiden, The Netherlands
[2] Biomedical Photonic Imaging, Faculty of Science and Technology, University of Twente, Enschede, The Netherlands
[3] Health Technology and Services Research, Faculty of Behavioural Management and Social Sciences, University of Twente, Enschede, The Netherlands
[4] Department of Internal Medicine, division Endocrinology, Leiden University Medical Centre, Leiden, The Netherlands
[5] Centre for Bone Quality Leiden, Leiden University Medical Centre, Leiden, The Netherlands
[6] Department of Nuclear Medicine, Centre François Baclesse, Caen, France
[7] Department of Surgery, Leiden University Medical Centre, Leiden, The Netherlands
[8] Department of Radiology, section Nuclear Medicine, Alrijne Medical Centre, Leiden, The Netherlands
[9] Department of Radiation Sciences and Technology, Delft University of Technology, Delft, The Netherlands
[10] Department of Medical Imaging, Radboud University Medical Centre, Nijmegen, The Netherlands

* Corresponding author: Ir. Sietse van Mossel (s.van_mossel@lumc.nl; 2333 ZA Leiden, The Netherlands)

Probabilities related to preoperative imaging performance

In this appendix details are provided on the performance of different imaging strategies used for simulation. For example, the base case probability of a negative MIBI SPECT/CT scan in current best practice is 0.204, the probability of a positive MIBI SPECT/CT scan in current best practice is 0.796, the probability of a positive [^{18}F]FCH PET/CT scan in current best practice is 0.760 and the probability of a negative [^{18}F]FCH PET/CT scan in current best practice is 0.240. It can be calculated that the probability of a patient receiving at least one positive scan is 0.796 + (0.204 * 0.760) = 95% and the probability of a patient receiving negative or inconclusive scans only is 5% in current best practice. In comparison, the probability of a positive [^{18}F]FCH PET/CT scan using the one-stop-shop strategy is also 95%. This is in line with [^{18}F]FCH PET/CT being the superior imaging technique for the localisation of parathyroid adenomas and according to several meta-analyses [1–6].

The yearly probability of receiving repeated imaging is 0.387 and exponentially distributed based on expert opinion. It is assumed that the medical specialist and patient yearly perform shared decision-making on whether or not to make a new scan based on the effect of pharmacotherapy and the clinical condition of the patient. If a scan has been made, it is assumed that the medical specialist has good reasons to do so and the probability of a positive test result is estimated to be similar to first-line imaging (probability of 95%).

Probabilities related to surgical management and pharmacotherapy

In this appendix details are also provided on the performance of the treatment strategies included for simulation. It is assumed that patients with a positive imaging test result receive parathyroidectomy and patients with a negative or inconclusive test result explorative neck dissection (ioPTH-monitored treatment setting) or pharmacotherapy and active surveillance (traditional treatment setting). Also, MIP can immediately be converted to explorative neck dissection after ioPTH monitoring (this does not hold for the traditional treatment setting). Base case probabilities of curation after surgical management are high. For example, the curation after MIP is 91%. If MIP is not curative, explorative neck dissection can be performed and the probability of curation after [MIP plus explorative neck dissection] is 0.914 + (1-0.914) * 0.506 = 96%. The probability of full curation after repeated parathyroidectomy is lower but still high (value of 87%). Furthermore, probabilities of complications due to surgical management are low (values smaller than 5%) and probabilities of suffering from hypoparathyroidism or hyperparathyroidism after surgical management are also low (values smaller than 5% and 10% respectively).

In case of non-curative surgical management, patients will experience persistent hyperparathyroidism and may suffer from secondary disorders. The accumulated probability of suffering from secondary disorders (such as neurological, cardiovascular, bone and renal events causing increased morbidity and decreased quality of life) is 0.246 (i.e., approximately one out of four patients on average).

Simulated parameter values, parametric distributions and corresponding data sources. All data sources are supplemented in this file, but also cited in the text of the main manuscript.

Parameter description	Source	Base case value [a]	Parameter distribution	Parameter 1	Parameter 2	Parameter explanation [b]
Age at diagnosis with PHPT	[7]	62	Normal [c]	62	14	Param 1 = mean; param 2 = stdev
Probability of suffering secondary disorders in case of hyperparathyroidism	[8]	0,246	Beta	37	113	Param 1 = alpha; param 2 = beta
Duration of a MIP procedure in minutes	[7]	40	Gamma	40	8	Param 1 = mean; param 2 = stdev
Duration of a blind neck exploration procedure in minutes	[7]	100	Gamma	100	20	Param 1 = mean; param 2 = stdev
Duration of a standard parathyroidectomy procedure in minutes	[7]	55	Gamma	55	11	Param 1 = mean; param 2 = stdev
Duration of ioPTH monitoring in minutes	Expert elicitation	30	Gamma	30	6	Param 1 = mean; param 2 = stdev
Probability that complications occur after (minimally invasive) parathyroidectomy	[9]	0,015	Beta	24	1626	Param 1 = alpha; param 2 = beta
Probability that complications occur after blind neck exploration	[9]	0,031	Beta	51	1599	Param 1 = alpha; param 2 = beta
Probability of a negative MIBI SPECT/CT scan in current best practice	[7]	0,204	Beta	951	3700	Param 1 = alpha; param 2 = beta
Probability of a positive MIBI SPECT/CT scan in current best practice	[7]	0,796	Beta	3700	951	Param 1 = alpha; param 2 = beta
Probability of a positive 18F-FCH PET/CT scan in current best practice	[10]	0,760	Beta	19	6	Param 1 = alpha; param 2 = beta
Probability of a negative 18F-FCH PET/CT scan in current best practice	[10]	0,240	Beta	6	19	Param 1 = alpha; param 2 = beta
Probability of a positive 18F-FCH PET/CT scan using the one-stop-shop strategy	[11]	0,951	Beta	131	8	Param 1 = alpha; param 2 = beta
Probability of a negative 18F-FCH PET/CT scan using the one-stop-shop strategy	[11]	0,049	Beta	8	131	Param 1 = alpha; param 2 = beta
Yearly probability of receiving repeated imaging	Expert elicitation	0,387	Exponential	1,053 [g]		Param 1 = rate
Probability that patients benefit from second-line imaging before repeating surgery	Expert elicitation	0,951 [g]	Beta	131	8	Param 1 = alpha; param 2 = beta

Probability of receiving MIP after a positive scan	Expert elicitation	1,000	Fixed	n/a	n/a	n/a
Probability of receiving repeated parathyroidectomy after a positive scan	Expert elicitation	1,000	Fixed	n/a	n/a	n/a
Probability of receiving blind neck exploration after a negative scan [d]	Expert elicitation	1,000	Fixed	n/a	n/a	n/a
Probability that MIP is converted to blind neck exploration after ioPTH monitoring	[7]	0,085	Beta	396	4287	Param 1 = alpha; param 2 = beta
Probability of suffering hypoparathyroidism after MIP	[7]	0,001	Beta	0,0009	0,0002	Param 1 = mean; param 2 = stdev
Probability of full curation after MIP	[7]	0,914	Beta	4287	396	Param 1 = alpha; param 2 = beta
Probability of suffering hyperparathyroidism after blind neck exploration	[7]	0,038	Beta	253	5608	Param 1 = alpha; param 2 = beta
Probability of suffering hypoparathyroidism after blind neck exploration	[7]	0,005	Beta	0,005	0,001	Param 1 = mean; param 2 = stdev
Probability of full curation after blind neck exploration (after MIP of a culprit lesion) [e]	[7]	0,506	Beta	5608	253	Param 1 = alpha; param 2 = beta
Probability of suffering persistent hyperparathyroidism after repeated parathyroidectomy	[7]	0,064	Beta	299	3232	Param 1 = alpha; param 2 = beta
Probability of suffering secondary disorders in case of persistent hyperparathyroidism	[7]	0,021	Beta	97	1055	Param 1 = alpha; param 2 = beta
Probability of suffering hypoparathyroidism after repeated parathyroidectomy	[7]	0,050	Beta	0,05	0,01	Param 1 = mean; param 2 = stdev
Probability of full curation after repeated parathyroidectomy	[7]	0,865	Beta	4287	396	Param 1 = alpha; param 2 = beta
Costs of biochemical testing including serum PTH (Nza tariff declaration code 072646)	[12]	7,55	Gamma	7,55	1,51	Param 1 = mean; param 2 = stdev
Costs of biochemical testing including calcium in faeces (Nza tariff declaration code 070212)	[12]	2,89	Gamma	2,89	0,58	Param 1 = mean; param 2 = stdev
Costs of cervical ultrasonography (Nza tariff declaration code 082970)	[12]	83,75	Gamma	83,75	16,75	Param 1 = mean; param 2 = stdev
Costs of abdominal ultrasonography (Nza tariff declaration code 087070)	[12]	92,42	Gamma	92,42	18,48	Param 1 = mean; param 2 = stdev
Costs of cervical contrast-enhanced CT imaging (Nza tariff declaration code 085042)	[12]	187,39	Gamma	187,39	37,48	Param 1 = mean; param 2 = stdev
Costs of urinary system CT imaging (Nza tariff declaration code 088012)	[12]	123,46	Gamma	123,46	24,69	Param 1 = mean; param 2 = stdev

Costs of planar scintigraphy of the parathyroid glands region (Nza tariff declaration code 120013)	[12]	237,42	Gamma	237,42	47,48	Param 1 = mean; param 2 = stdev
Costs of MIBI SPECT/CT scan of the thorax (Nza tariff declaration code 120280)	[12]	350,10	Gamma	350,10	70,02	Param 1 = mean; param 2 = stdev
Costs of partial body PET with FCH as radiopharmaceutical (Nza tariff declaration code 120500)	[12]	965,93	Gamma	965,93	193,19	Param 1 = mean; param 2 = stdev
Costs of a bone density scan with DEXA resources (Nza tariff declaration code 120032)	[12]	121,02	Gamma	121,02	24,20	Param 1 = mean; param 2 = stdev
Costs of surgeon during patient-specific interventions per hour in Dutch hospitals	[13]	137,23	Gamma	113,00	22,60	Param 1 = mean; param 2 = stdev
Costs of hospitalisation in Dutch hospitals per day	[13]	779,68	Gamma	642,00	128,40	Param 1 = mean; param 2 = stdev
Costs of an outpatient consultation session in Dutch hospitals	[13]	197,96	Gamma	163,00	32,60	Param 1 = mean; param 2 = stdev
Costs of outpatient care	[13]	335,19	Gamma	276,00	55,20	Param 1 = mean; param 2 = stdev
Costs of blind neck exploration (Nza tariff declaration code 029699005)	[12]	5488,06	Gamma	5488,06	1097,61	Param 1 = mean; param 2 = stdev
Costs of MIP (Nza tariff declaration code 029699005)	[12]	2195,22	Gamma	2195,22	439,04	Param 1 = mean; param 2 = stdev
Costs of parathyroidectomy (Nza tariff declaration code 029699005)	[12]	3018,43	Gamma	3018,43	603,69	Param 1 = mean; param 2 = stdev
Surgical costs of treating vertebral fractures (Nza tariff declaration code 199299124)	[12]	7193,85	Gamma	7193,85	1438,77	Param 1 = mean; param 2 = stdev
Lithotripsy costs of treating renal stones (Nza tariff declaration code 140401015)	[12]	1242,27	Gamma	1242,27	248,45	Param 1 = mean; param 2 = stdev
Costs of supplement alendronic acid (70 mg) as standard bisphosphonate option	[14]	0,25	Gamma	0,25	0,05	Param 1 = mean; param 2 = stdev
Costs of supplement cinacalcet (30 mg) as standard cinacalcet option	[14]	4,60	Gamma	4,60	0,92	Param 1 = mean; param 2 = stdev
Costs of supplement hydrochlorothiazide (50 mg) as standard diuretics options	[14]	0,05	Gamma	0,05	0,01	Param 1 = mean; param 2 = stdev
Costs of injection zoledronic acid (0.8 mg) as part of osteoporosis outpatient care	[14]	119,75	Gamma	119,75	23,95	Param 1 = mean; param 2 = stdev
Costs of supplement calcium/vitamin D3 (500mg) in case of hypoparathyroidism	[14]	0,11	Gamma	0,11	0,02	Param 1 = mean; param 2 = stdev
Costs of supplement alfacalcidol (0.75 mcg) in case of hypoparathyroidism	[14]	0,46	Gamma	0,46	0,09	Param 1 = mean; param 2 = stdev

Costs of injection teriparatide (250 mcg) as alternative in case of hypoparathyroidism	[14]	299,00	Gamma	299,00	59,80	Param 1 = mean; param 2 = stdev
Utility after curation and remaining in full health	[15]	0,839	Beta	0,839	0,179	Param 1 = mean; param 2 = stdev
Disutility for experiencing complications after surgical management	[15]	0,179	Gamma	0,179	0,036	Param 1 = mean; param 2 = stdev
Utility when experiencing long-term hypoparathyroidism	[16,17]	0,778	Beta	0,778	0,156	Param 1 = mean; param 2 = stdev
Utility when experiencing primary, recurrent or persistent hyperparathyroidism [f]	[18]	0,839	Beta	0,839	0,179	Param 1 = mean; param 2 = stdev
Utility when experiencing secondary disorder(s) as a consequence of hyperparathyroidism [f]	[19,20]	0,590	Beta	0,590	0,118	Param 1 = mean; param 2 = stdev

[a] All estimated cost values are in Euros ("€") and year of conversion is 2024, respectively.

[b] If no information was available on the uncertainty of cost and utility parameter values, we simulated ±20% base case value as an estimate for the standard deviation.

[c] Normally distributed age parameter is truncated for negative values.

[d] Blind neck exploration is provided in the ioPTH-monitored treatment setting, while in the traditional treatment setting pharmacotherapy with active surveillance is provided.

[e] Here, the following calculation should be made to estimate the probability of curation after MIP *plus* explorative neck dissection: 0.914 + (1-0.914) * 0.506 = 96%. Thus, the probability of being cured after surgical management including MIP and sequentially (i.e., with immediate conversion to) explorative neck dissection is 96%.

[f] Utility of a patient experiencing hyperparathyroidism is not significantly lower than a patient in full health, but a patient's utility is decreasing substantially when experiencing secondary disorders – such as neurological, cardiovascular, bone and renal events – as a consequence of hyperparathyroidism.

[g] The exponential distribution parameter value is determined based on medical specialists' expertise that repeated imaging is provided each 2-3 years on average. It is assumed that medical specialists have good reasons to provide repeated imaging and, thus, the probability of a positive test result is estimated to be similar to first imaging.

References

1. Treglia G, Piccardo A, Imperiale A, Strobel K, Kaufmann PA, Prior JO, et al. Diagnostic performance of choline PET for detection of hyperfunctioning parathyroid glands in hyperparathyroidism: a systematic review and meta-analysis. Eur J Nucl Med Mol Imaging. 2019;46:751–65.

2. Evangelista L, Ravelli I, Magnani F, Maurizio Iacobone ·, Chiara Giraudo ·, Camozzi V, et al. 18F-choline PET/CT and PET/MRI in primary and recurrent hyperparathyroidism: a systematic review of the literature. Ann Nucl Med. 2020;34:601–19.

3. Broos WAM, Van Der Zant FM, Knol RJJ, Wondergem M. Choline PET/CT in parathyroid imaging: a systematic review. Nucl Med Commun. 2019;40:96–105.

4. Kluijfhout WP, Pasternak JD, Drake FT, Beninato T, Gosnell JE, Shen WT, et al. Use of PET tracers for parathyroid localization: a systematic review and meta-analysis. Langenbecks Arch Surg. 2016;401:925–35.

5. Whitman J, Allen IE, Bergsland EK, Suh I, Hope TA. Assessment and Comparison of 18F-Fluorocholine PET and 99mTc-Sestamibi Scans in Identifying Parathyroid Adenomas: A Metaanalysis. Journal of Nuclear Medicine. 2021;62:1285–91.

6. Lee SW, Shim SR, Jeong SY, Kim SJ. Direct Comparison of Preoperative Imaging Modalities for Localization of Primary Hyperparathyroidism: A Systematic Review and Network Meta-analysis. JAMA Otolaryngology–Head & Neck Surgery. 2021;147:692–706.

7. Bergenfelz A, Van Slycke S, Makay Ö, Brunaud L. European multicentre study on outcome of surgery for sporadic primary hyperparathyroidism. British Journal of Surgery. 2021;108:675–83.

8. Vignali E, Viccica G, Diacinti D, Cetani F, Cianferotti L, Ambrogini E, et al. Morphometric vertebral fractures in postmenopausal women with primary hyperparathyroidism. Journal of Clinical Endocrinology and Metabolism. 2009;94:2306–12.

9. Udelsman R, Lin Z, Donovan P. The superiority of minimally invasive parathyroidectomy based on 1650 consecutive patients with primary hyperparathyroidism. Ann Surg. 2011;253:585–91.

10. Quak E, Blanchard D, Houdu B, Le Roux Y, Ciappuccini R, Lireux B, et al. F18-choline PET/CT guided surgery in primary hyperparathyroidism when ultrasound and MIBI SPECT/CT are negative or inconclusive: the APACH1 study. Eur J Nucl Med Mol Imaging. 2018;45:658–66.

11. Broos WAM, Wondergem M, Knol RJJ, van der Zant FM. Parathyroid imaging with 18F-fluorocholine PET/CT as a first-line imaging modality in primary hyperparathyroidism: a retrospective cohort study. EJNMMI Res. 2019;9:72.

12. Dutch Healthcare Authority. Dutch dbc-information system. https://www.opendisdata.nl. Accessed August 25. 2023.

13. Dutch Healthcare Authority. Richtlijn voor het uitvoeren van economische evaluaties in de gezondheidzorg. https://www.zorginstituutnederland.nl/publicaties/publicatie/2016/02/29/richtlijn-voor-het-uitvoeren-van-economische-evaluaties-in-de-gezondheidszorg. Accessed February 5. 2024.

14. Dutch Healthcare authority. Dutch drug database. https://www.medicijnkosten.nl. Accessed August 25. 2023.

15. Versteegh M, M. Vermeulen K, M. A. A. Evers S, de Wit GA, Prenger R, A. Stolk E. Dutch Tariff for the Five-Level Version of EQ-5D. Value in Health. 2016;19:343–52.

16. Vokes T. Quality of life in hypoparathyroidism. Bone. 2019;120:542–7.

17. Astor MC, Løvas K, Debowska A, Eriksen EF, Evang JA, Fossum C, et al. Epidemiology and health-related quality of life in hypoparathyroidism in Norway. Journal of Clinical Endocrinology and Metabolism. 2016;101:3045–53.

18. Ye Z, Silverberg SJ, Sreekanta A, Tong K, Wang Y, Chang Y, et al. The Efficacy and Safety of Medical and Surgical Therapy in Patients With Primary Hyperparathyroidism: A Systematic Review and Meta-Analysis of Randomized Controlled Trials. Journal of Bone and Mineral Research. 2022;37:2351–72.

19. Lips P, Cooper C, Agnusdei D, Caulin F, Egger P, Johnell O, et al. Quality of Life in Patients with Vertebral Fractures: Validation of the Quality of Life Questionnaire of the European Foundation for Osteoporosis (QUALEFFO) Osteoporosis International. 1999.

20. Tapiero S, Limfuco L, Bechis SK, Sur RL, Penniston KL, Nakada SY, et al. The impact of the number of lifetime stone events on quality of life: results from the North American Stone Quality of Life Consortium. Urolithiasis. 2021;49:321–6.
