# Supplementary material for: Cost-effectiveness of one-stop-shop [18F]Fluorocholine PET/CT to localise parathyroid adenomas in patients suffering from primary hyperparathyroidism
Source: Eur J Nucl Med Mol Imaging. 2024 Jun 5;51(12):3585–95. doi: 10.1007/s00259-024-06771-1 (PMC11457719; doi:10.1007/s00259-024-06771-1)
Supplement: Supplementary file 4 — Supplementary file4 (PDF 712 KB) [file 259_2024_6771_MOESM4_ESM.pdf]

# Cost-Effectiveness of One-Stop-Shop [$^{18}$F]Fluorocholine PET/CT to Localise Parathyroid Adenomas in Patients Suffering from Primary Hyperparathyroidism

*European Journal of Nuclear Medicine and Molecular Imaging (EJNMMI)*

Sietse van Mossel [1,2,*], Sopany Saing [3], Natasha Appelman-Dijkstra [4,5], Elske Quak [6], Abbey Schepers [7], Frits Smit[1,8], Lioe-Fee de Geus-Oei [1,2,9], Dennis Vriens [1,5,10]

[1] Department of Radiology, section Nuclear Medicine, Leiden University Medical Centre, Leiden, The Netherlands

[2] Biomedical Photonic Imaging, Faculty of Science and Technology, University of Twente, Enschede, The Netherlands

[3] Health Technology and Services Research, Faculty of Behavioural Management and Social Sciences, University of Twente, Enschede, The Netherlands

[4] Department of Internal Medicine, division Endocrinology, Leiden University Medical Centre, Leiden, The Netherlands

[5] Centre for Bone Quality Leiden, Leiden University Medical Centre, Leiden, The Netherlands

[6] Department of Nuclear Medicine, Centre François Baclesse, Caen, France

[7] Department of Surgery, Leiden University Medical Centre, Leiden, The Netherlands

[8] Department of Radiology, section Nuclear Medicine, Alrijne Medical Centre, Leiden, The Netherlands

[9] Department of Radiation Sciences and Technology, Delft University of Technology, Delft, The Netherlands

[10] Department of Medical Imaging, Radboud University Medical Centre, Nijmegen, The Netherlands

* Corresponding author: Ir. Sietse van Mossel (s.van_mossel@lumc.nl; 2333 ZA Leiden, The Netherlands)

**(Supplementary Information) Fig. 1** Cost-effectiveness planes for the ioPTH-monitored and traditional treatment setting. The cost-effectiveness planes show the results of the Monte Carlo experiments. The figure shows the (**a-b**) ioPTH-monitored and (**c-d**) traditional treatment setting. In both treatment settings (**a**,**c**), the experiments suggest that the total expected costs and health effects per patient were similar for the one-stop-shop strategy (orange point estimates) and current best practice (black point estimates). Accordingly, the mean cost and effectiveness for each imaging strategy were not materially different (orange and black spheres are overlapping). Furthermore, the incremental planes (**b**,**d**) show that not only the mean cost and effectiveness for each imaging strategy were similar but also the individual point estimates. QALY increments were approximately zero while cost increments varied between -€200 and €300. Abbreviations: ioPTH, intraoperative parathyroid hormone serum level. WTP, willingness-to-pay. QALY, quality-adjusted life year.

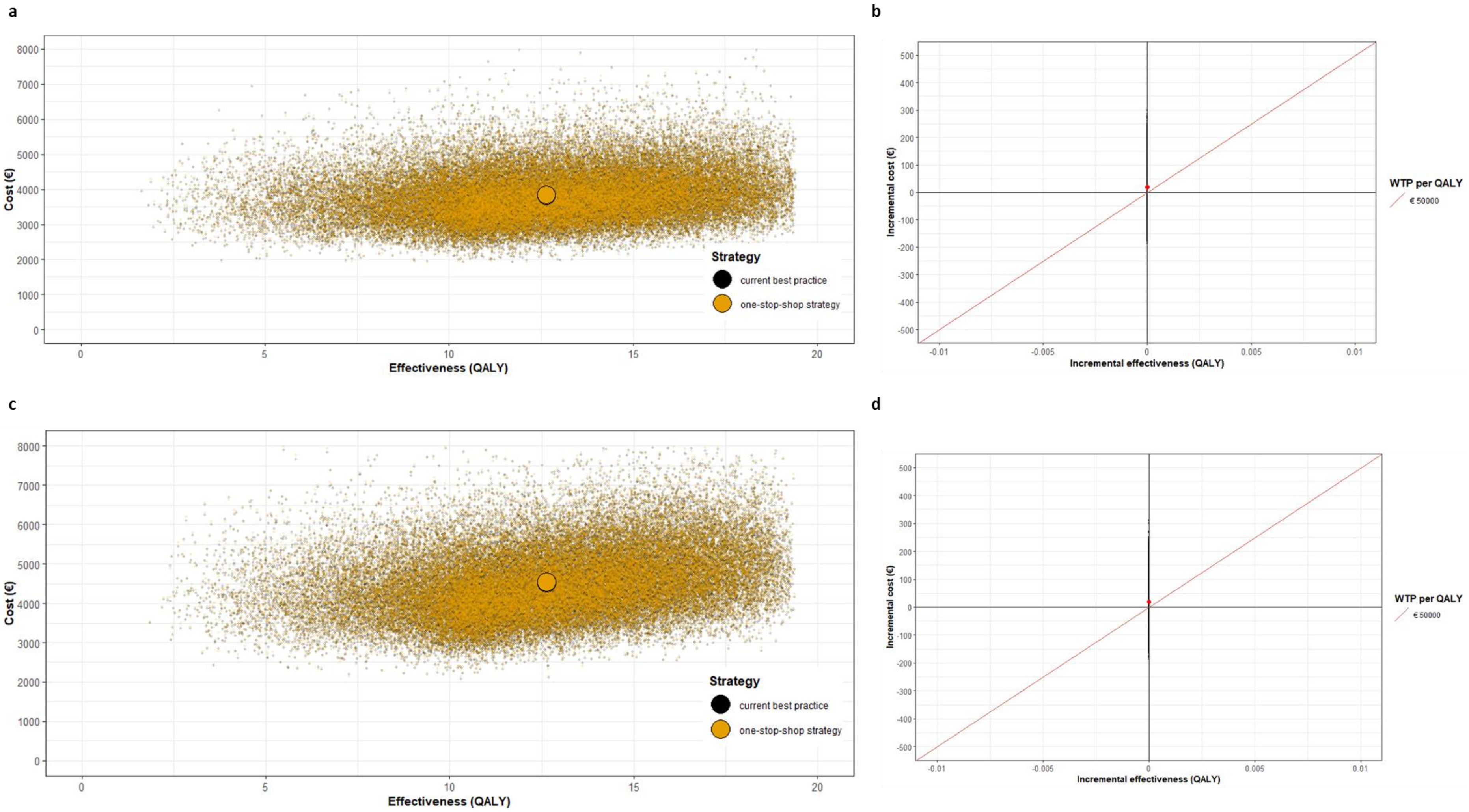
a
b
c
d
Cost (€)
Effectiveness (QALY)
Strategy
current best practice
one-stop-shop strategy
Incremental cost (€)
Incremental effectiveness (QALY)
WTP per QALY
€ 50000

**(Supplementary Information) Fig. 2** Given the traditional treatment setting, decision curves depicting **(a)** the tariff of [$^{18}$F]FCH PET/CT on the x-axis and **(b)** the sensitivity of MIBI SPECT/CT on the x-axis. The total costs including imaging, surgery and pharmacotherapy of the expected PHPT care pathway are depicted on the y-axis and given a traditional treatment setting. Abbreviations: [$^{18}$F]FCH PET/CT, positron emission tomography and computed tomography using [$^{18}$F]Fluorocholine. MIBI SPECT/CT, single-photon emission computed tomography and computed tomography using [$^{99m}$Tc]Tc-methoxy isobutyl isonitrile. PHPT, primary hyperparathyroidism.

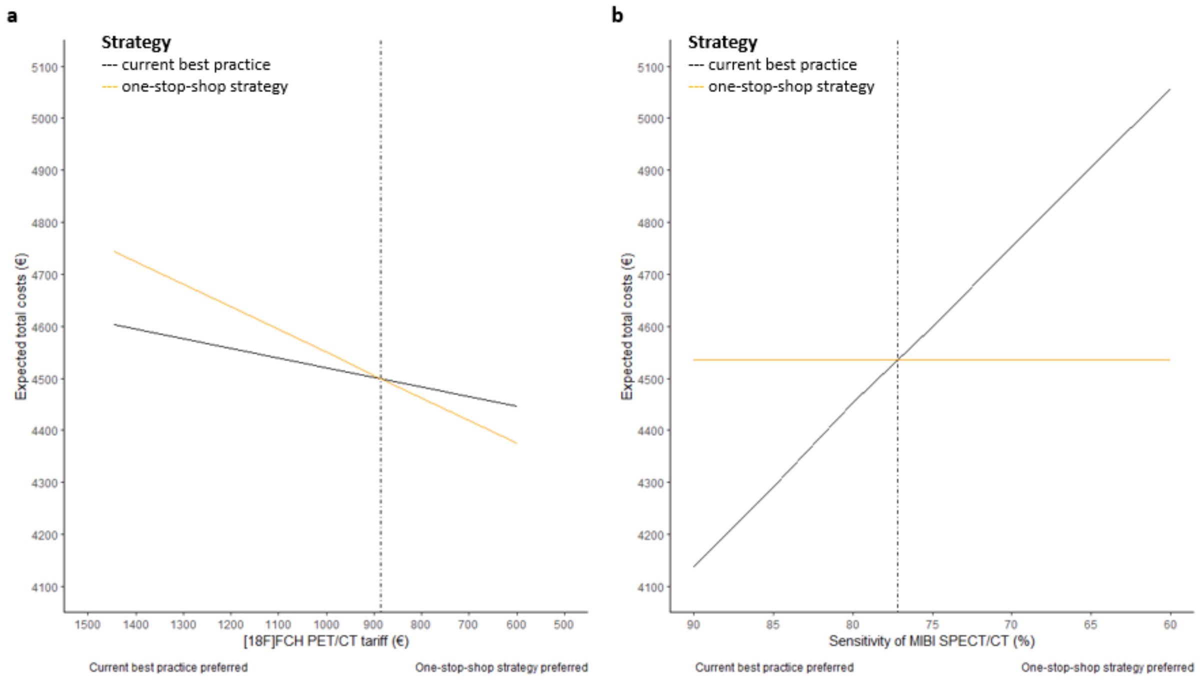

**(Supplementary Information) Fig. 3** Tornado diagram of the one-way sensitivity analysis performed. Tornado diagram of the one-way sensitivity analyses with 95% confidence interval ranges for all model parameters compared to the base case. The tornado diagram depicts the input parameters that lead to a relative change in the incremental NMB of at least 10% compared to the base case. The tornado diagram is based on a polynomial regression metamodel of the base case analysis. Therefore, the incremental NMB of current best practice compared to the one-stop-shop strategy shown in the plot (approximately €30) is slightly different than the simulated incremental NMB (approximately €0). The tornado diagram highlights the sensitivity of the cost of partial-body [$^{18}$F]FCH PET/CT: the cost of partial-body [$^{18}$F]FCH PET/CT has the most impact on the incremental NMB. If the cost of [$^{18}$F]FCH PET/CT increases, the incremental NMB of current best practice increases. This holds for both treatment settings. The consequences of a discounted cost of [$^{18}$F]FCH PET/CT are not depicted because we performed a separate threshold analysis. Abbreviations: NMB, net monetary benefit. US, ultrasonography. SPECT/CT, single-photon emission computed tomography and computed tomography using [$^{99m}$Tc]Tc-methoxy isobutyl isonitrile. PET/CT, positron emission tomography and computed tomography using [$^{18}$F]Fluorocholine.

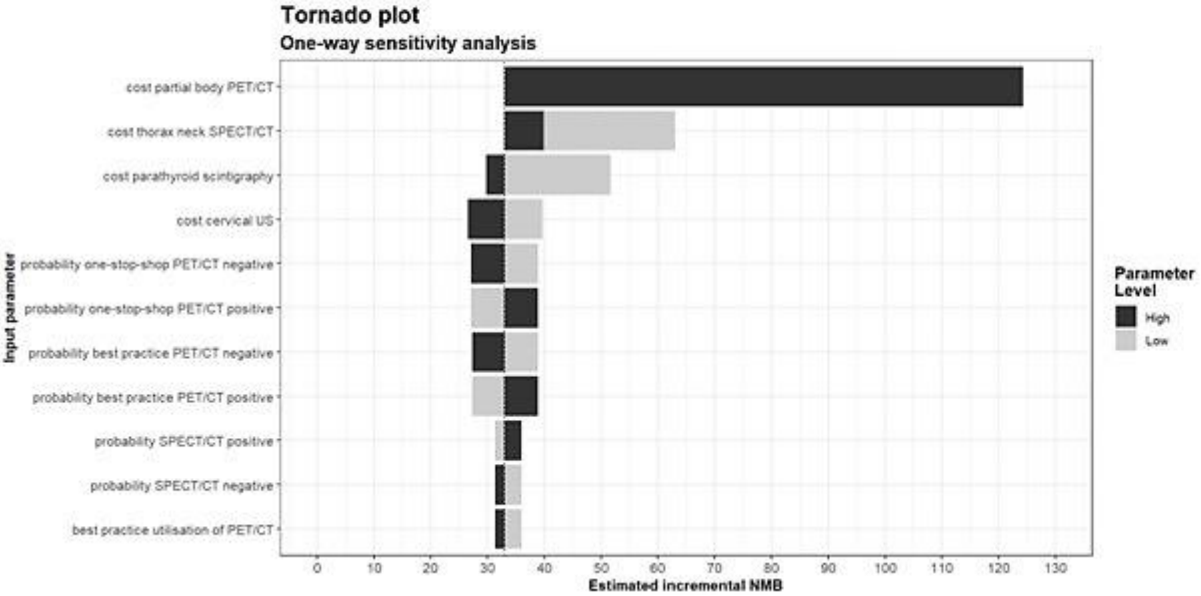

**(Supplementary Information) Fig. 4** Given the traditional treatment setting, the cost-effectiveness acceptability curves show the probability that the one-stop-shop strategy is cost-effective compared to current best practice at different WTP thresholds per QALY ranging from €0 to €100,000. The first curve (**a**) shows that incorporating a fixed disutility of 0.005, as a consequence of radiation burden for patients receiving MIBI SPECT/CT and [$^{18}$F]FCH PET/CT, results in an ICER of €49,909 and a 51% probability that the one-stop-shop strategy is cost-effective at the most accepted WTP threshold of €50,000 per QALY. The second curve (**b**) shows that incorporating a fixed disutility of 0.01, as a consequence of challenging logistics for patients receiving three different preoperative scans, results in an ICER of €24,954 and a 65% probability that the one-stop-shop strategy is cost-effective at the most accepted WTP threshold of €50,000 per QALY. Abbreviations: WTP, willingness-to-pay. QALY, quality-adjusted life year. ICER, incremental cost-effectiveness ratio. [$^{18}$F]FCH PET/CT, positron emission tomography and computed tomography using [$^{18}$F]Fluorocholine. MIBI SPECT/CT, single-photon emission computed tomography and computed tomography using [$^{99m}$Tc]Tc-methoxy isobutyl isonitrile.

a

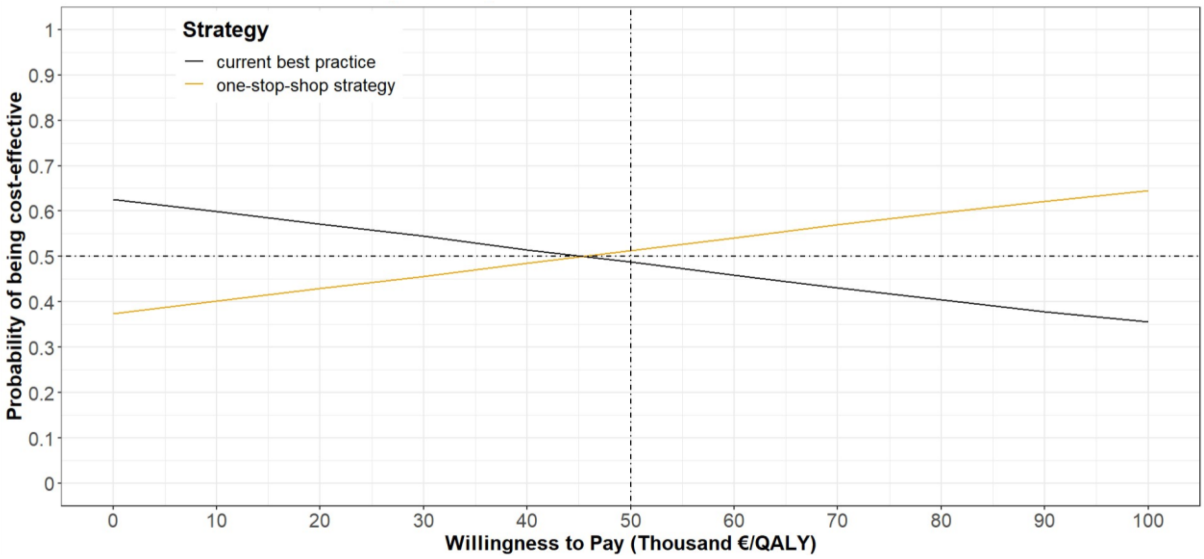
Strategy
current best practice
one-stop-shop strategy
Probability of being cost-effective
1
0.9
0.8
0.7
0.6
0.5
0.4
0.3
0.2
0.1
0
0
10
20
30
40
50
60
70
80
90
100
Willingness to Pay (Thousand €/QALY)

b

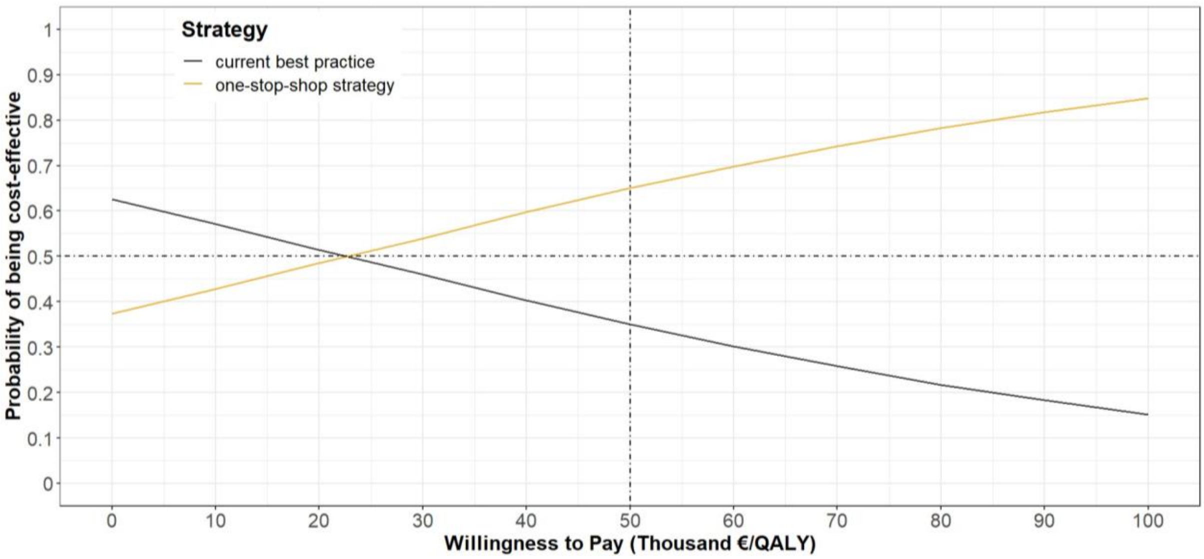
Strategy
current best practice
one-stop-shop strategy
Probability of being cost-effective
1
0.9
0.8
0.7
0.6
0.5
0.4
0.3
0.2
0.1
0
0
10
20
30
40
50
60
70
80
90
100
Willingness to Pay (Thousand €/QALY)
