# Supplementary material for: Cost-effectiveness of one-stop-shop [18F]Fluorocholine PET/CT to localise parathyroid adenomas in patients suffering from primary hyperparathyroidism
Source: Eur J Nucl Med Mol Imaging. 2024 Jun 5;51(12):3585–95. doi: 10.1007/s00259-024-06771-1 (PMC11457719; doi:10.1007/s00259-024-06771-1)
Supplement: Supplementary file 5 — Supplementary file5 (PDF 786 KB) [file 259_2024_6771_MOESM5_ESM.pdf]

Cost-Effectiveness of One-Stop-Shop [^{18}F]Fluorocholine PET/CT to Localise Parathyroid Adenomas in Patients Suffering from Primary Hyperparathyroidism

European Journal of Nuclear Medicine and Molecular Imaging (EJNMMI)

Sietse van Mossel [1,2,*], Sopany Saing [3], Natasha Appelman-Dijkstra [4,5], Elske Quak [6], Abbey Schepers [7], Frits Smit[1,8], Lioe-Fee de Geus-Oei [1,2,9], Dennis Vriens [1,5,10]

[1] Department of Radiology, section Nuclear Medicine, Leiden University Medical Centre, Leiden, The Netherlands
[2] Biomedical Photonic Imaging, Faculty of Science and Technology, University of Twente, Enschede, The Netherlands
[3] Health Technology and Services Research, Faculty of Behavioural Management and Social Sciences, University of Twente, Enschede, The Netherlands
[4] Department of Internal Medicine, division Endocrinology, Leiden University Medical Centre, Leiden, The Netherlands
[5] Centre for Bone Quality Leiden, Leiden University Medical Centre, Leiden, The Netherlands
[6] Department of Nuclear Medicine, Centre François Baclesse, Caen, France
[7] Department of Surgery, Leiden University Medical Centre, Leiden, The Netherlands
[8] Department of Radiology, section Nuclear Medicine, Alrijne Medical Centre, Leiden, The Netherlands
[9] Department of Radiation Sciences and Technology, Delft University of Technology, Delft, The Netherlands
[10] Department of Medical Imaging, Radboud University Medical Centre, Nijmegen, The Netherlands

* Corresponding author: Ir. Sietse van Mossel (s.van_mossel@lumc.nl; 2333 ZA Leiden, The Netherlands)

A third imaging strategy: the results of sequential US and [^{18}F]FCH PET/CT

In this separate simulation, we slightly adjusted the one-stop-shop strategy by including preoperative US. As described in the 2021 European Association of Nuclear Medicine (EANM) practice guidelines for parathyroid imaging, US provides an additional evaluation of the thyroid that might change patient management, especially in the case of coexisting (suspected) malignant nodules [1].

(Supplementary Information) Fig. 1 The results of the Monte Carlo experiments were plotted in cost-effectiveness planes. For the ioPTH-monitored treatment setting (Figure 1a), the adjusted one-stop-shop strategy had an estimated mean total cost of €3,874 per patient. Note that current best practice had an estimated mean total cost of €3,822 per patient (mean difference of €52). There was also no expected clinically relevant difference in the QALYs obtained as the estimated mean total QALY was 12.65 per patient in both strategies. For the traditional treatment setting (Figure 1b), the adjusted one-stop-shop strategy had an estimated mean total cost of €4,571 per patient. Note that current best practice had an estimated mean total cost of €4,514 per patient (mean difference of €57). There was also no expected clinically relevant difference in the QALYs obtained as the estimated mean total QALY was 12.64 per patient in both strategies. Indeed, when US is omitted, coincidental thyroid disorders might be overlooked in a subset of patients suffering from PHPT. However, a clinically relevant difference in the expected QALYs was not expected (i.e., the subset of patients with a thyroid disorder and having a decreased life expectancy because of this disorder was expected to be very small).

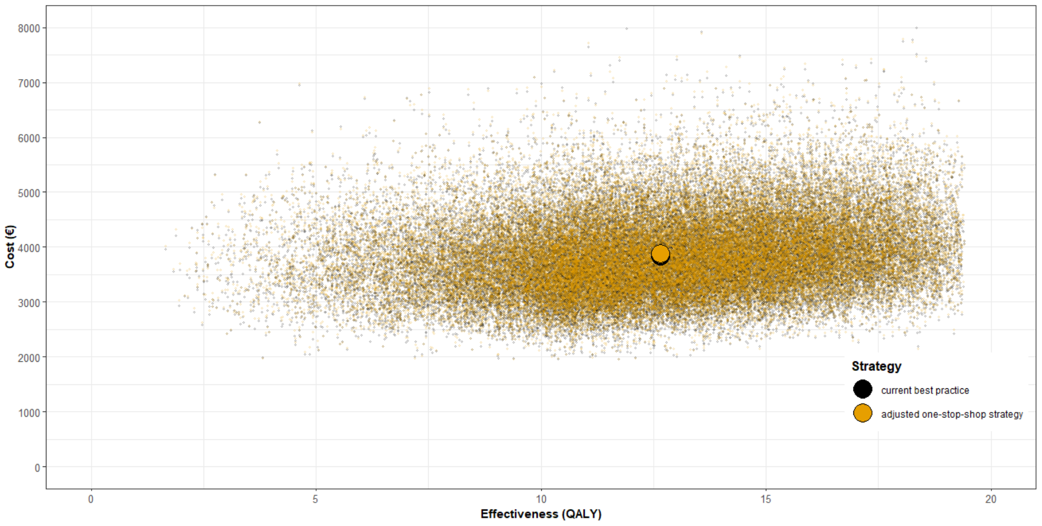

(a)

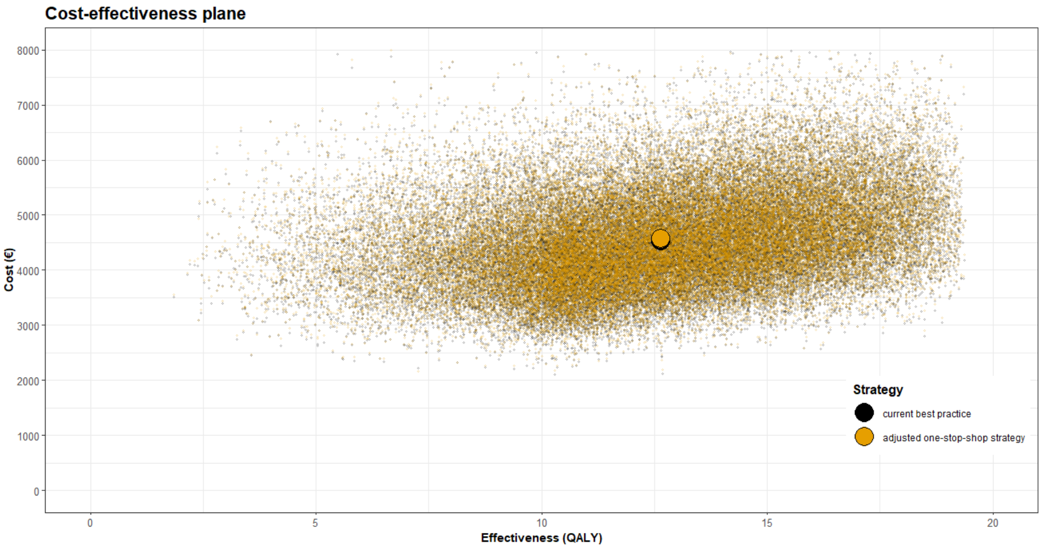

(b)

(Supplementary Information) Fig. 2 Decision curves show the results of the threshold analyses for the ioPTH-monitored and traditional treatment setting. Given the ioPTH-monitored treatment setting, Figure 3a shows that the adjusted one-stop-shop strategy was cost-effective when the base case tariff of partial-body [^{18}F]FCH PET/CT (€965) decreased by at least €290 (30%) resulting in a suggested tariff of €675. Respectively, Figure 3b shows that the adjusted one-stop-shop strategy was cost-effective when the base case sensitivity of MIBI SPECT/CT (79.6%) decreased to sensitivity values lower than 75.5%. Given the traditional treatment setting, Figure 3c shows that the adjusted one-stop-shop strategy was cost-effective when the base case tariff of partial-body [^{18}F]FCH PET/CT (€965) decreased by at least €305 (32%) resulting in a suggested tariff of €660. Respectively, Figure 3d shows that the adjusted one-stop-shop strategy was cost-effective when the base case sensitivity of MIBI SPECT/CT (79.6%) decreased to sensitivity values lower than 75.2%.

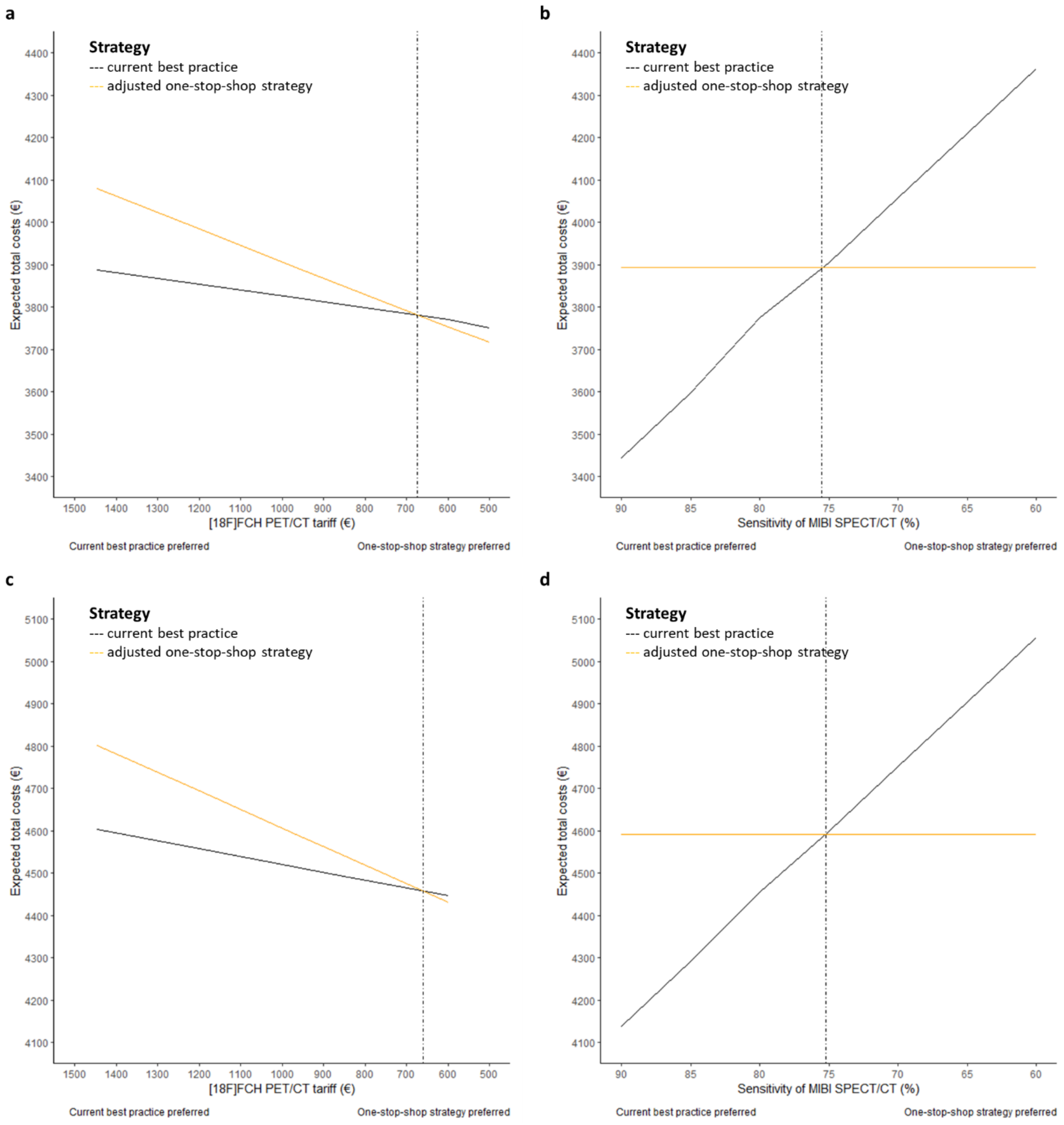

References

1. Ovčariček PP, Giovanella L, Gasset IC, Hindié E, Huellner MW, Luster M, et al. The EANM practice guidelines for parathyroid imaging. Eur J Nucl Med Mol Imaging. 2021;48:2801–22.
